# Change Adaptation Resilience Evaluation Questionnaire for Industry 5.0 Among Workers in High-Tech Manufacturing Companies: Questionnaire Development and Pilot Evaluation Study

**DOI:** 10.2196/83754

**Published:** 2026-08-28

**Authors:** Giulia Bassi, Angelo Valente, Serena Tassoni, Silvia Salcuni

**Affiliations:** 1 Department of Developmental and Socialization Psychology, University of Padova, Via Venezia 8, Padova, 35131, Italy, 39 3927534247

**Keywords:** resilience, technology, workers, Industry 5.0, manufacturing, instrument, pilot evaluation

## Abstract

**Background:**

Despite increasing recognition of human factors in technologically advanced manufacturing, psychological resilience remains an underexplored dimension. Existing studies often rely on generic instruments that fail to capture the specific adaptive challenges faced by workers in high-tech environments. There is a growing need for context-sensitive tools capable of assessing resilience in line with the human-centric vision of Industry 5.0.

**Objective:**

This study aimed to develop and conduct a preliminary evaluation of the Change Adaptation Resilience Evaluation Questionnaire for Industry 5.0 (CARE-QI 5.0), a multidimensional instrument designed to assess individual and contextual factors contributing to psychological resilience among workers in high-tech manufacturing settings.

**Methods:**

The questionnaire was developed through a 4-phase process: literature review, expert evaluation, focus groups with operators, and pilot testing within 2 manufacturing companies. CARE-QI 5.0 conceptualizes resilience as a higher-order construct comprising 2 second-order dimensions—individual resilience and contextual resilience—further articulated into 18 first-order subscales. Internal consistency and intersubscale correlations were assessed using data from a sample of workers employed in the mechanical and packaging industries in Northeastern Italy.

**Results:**

Findings on the 84 items showed good internal consistency across all subscales and meaningful patterns of intercorrelation. External validity showed that problem-solving self-efficacy, cognitive flexibility, and problem-oriented coping were positively associated with both individual-based resilience and a wide range of contextual resources, including support given by organizations, colleagues, and family, as well as openness to change. In contrast, perseverance in the face of difficulties was linked to cognitive inflexibility, avoidance, and seeking change, suggesting the presence of rigid or less adaptive coping functioning.

**Conclusions:**

CARE-QI 5.0 provides a theoretically grounded and context-sensitive tool for assessing psychological resilience as a multidimensional construct in technologically evolving manufacturing environments. By integrating both individual and contextual protective resources, this instrument captures key adaptive processes relevant to Industry 5.0 scenarios, where human-technology interaction plays a central role. While further psychometric validation is needed, including confirmatory factor analysis and measurement invariance testing, CARE-QI 5.0 shows promise for both theoretical and research use. It can support organizations in monitoring workers’ adaptability and well-being, guiding targeted interventions and training strategies that align with workers’ resilience profiles and their technological experience.

## Introduction

### Background

The manufacturing field constantly faces multiple challenges arising from the elevated production standards imposed by the global market, which increasingly demand high levels of customization and product quality [1]. Within this scenario, the integration of advanced industrial automation and collaborative robotics technologies (eg, cobots) constitutes one of the most significant transformations introduced by Industry 4.0, aimed at enhancing production efficiency through smart and interconnected systems [2]. Despite these technological advancements, manufacturing industries continue to encounter substantial barriers, including difficulties in technological implementation, as well as insufficient human worker integration into automated systems [3,4]. This limited human-technology integration obstructs the shift toward more adaptive and resilient manufacturing environments, where human-robot interaction (HRI) is expected to play a key role in improving operational flexibility, safety, and decision-making processes [5]. In this context, the human contribution in manufacturing production systems remains a critical factor in shaping production performance and machinery effectiveness [6]. A growing body of literature displays the pivotal mediating role played by Human Capital in technologically advanced firms, facilitating the maximization of both labor output and productivity [7,8]. Concurrently, the effectiveness of human-robot collaboration (HRC) has been strongly linked to increased individual performance, which is largely supported by key human factors [9]. In this context, the development of a new theoretical framework on HRI is considered essential to fostering production systems that are not only more efficient but also more flexible and resilient [10]. Within this evolving paradigm, the role of workers has become increasingly central not only in supporting long-term economic growth through efficient production practices but also in activating resilience strategies that support organizations to recover from potential disruptions [11]. In response to these emerging needs, Industry 5.0 suggests a fundamental shift from a system-centered to a human-centered perspective, keeping the HRI at the core of a collaborative process model [5]. This human-centric vision further emphasizes the importance of human well-being and the preservation of a supportive working environment by promoting the principles of sustainability and resilience [12,13]. Among these principles, resilience stands out as a critical benchmark, defined as the capability to adapt and flexibly cope with vulnerabilities and disruptive changes while maintaining industrial productivity [12]. In this framework, manufacturing workers emerge as the key to such resilience. Through their involvement in HRI processes, they become pivotal agents in supporting both production efficiency and organizational adaptability [14]. To optimize productivity and efficiency within complex industrial environments, several studies converge on the idea that Industry 5.0 can holistically improve workers’ well-being and safety by integrating advanced technologies and a human-centered approach [15-17]. This perspective gives rise to the concept of Operator 5.0, a professional figure who goes beyond mere technical skills, by embracing a self-resilience–oriented mindset. Within this framework, *psychological resilience* refers to the individual’s ability to emotionally cope with crises and stressors, often supported by advanced technological tools, whereas *cognitive resilience* refers to the operator’s ability to sustain mental focus under stressful conditions, thereby reducing the likelihood of error during production processes [18]. Accordingly, Operator 5.0 is not merely a user of technology but also a carrier of psychological resources essential to flourish in hybrid HRC settings, making resilience a key competence in the definition of the Industry 5.0 paradigm.

Recent research examines various dimensions of manufacturing resilience, with particular emphasis on systemic and technological resilience. Nonetheless, despite the increasing recognition of human factors, psychological resilience remains a relatively underexplored area within manufacturing contexts [19]. Existing studies rely on non–sector-specific assessment tools and tend to lack longitudinal or mixed methods designs, which limits their ability to adequately capture the dynamic and evolving nature of resilience in high-tech workplaces [20]. This reveals a crucial gap in the current literature, particularly concerning the potential impact of Industry 5.0 technologies, such as collaborative robots, on workers’ psychological resilience. Addressing this gap is pivotal to developing more integrated and context-sensitive assessment frameworks that not only promote human well-being but also enhance workplace adaptability and responsiveness in increasingly manufacturing settings.

### Theoretical Background and Current Research Gaps in Resilience at Work

Although various theoretical perspectives on resilience have been proposed, the concept is widely understood as a dynamic and evolving process shaped by individuals’ ongoing interactions with their environment [21]. Despite the absence of unanimous consensus upon definition, conceptualization, and measurement frameworks, most assumptions in the literature underline 2 foundational concepts: adversity, typically defined as negative events or circumstances quantitatively associated with adaptive difficulties, and positive outcomes, understood as the capacity to recover or exceed prior levels of psychological functioning following adversity [22]. The American Psychological Association [23] defines resilience as “the ability to adapt in the face of trauma, adversity, tragedy or even significant ongoing stressors.” Among the personal resources that support resilient functioning in complex and evolving work environments, 2 constructs have emerged as particularly relevant: change adaptation, the perceived ability to effectively respond to and integrate workplace changes [24], and cognitive flexibility, the capacity to adjust mental strategies and reframe stressful events [25,26]. Both have been linked to improved emotional regulation and adaptive behavior under pressure [26] and can be considered essential components of resilience in technological contexts. Within this framework, protective factors play a fundamental role in promoting resilience by increasing the likelihood of a positive adaptation following exposure to stressors [27]. According to Windle [28], protective factors are pivotal in defining resilience and are typically classified into 2 categories. Internal positive factors refer to intraindividual characteristics that foster resilience and are specific to each individual [29]. These include several traits, such as positive self-perception, self-regulation, cognitive abilities, adaptive coping strategies, and stress inoculation [27,30]. In contrast, external positive factors encompass supportive social and environmental conditions derived from an individual’s immediate surroundings [29], such as strong social support (eg, positive relationships from family and friends) and community cohesion [27,30]. These resources affect how individuals perceive and deal with challenges, especially in work-related settings [31].

Increasingly, the literature suggests that resilience cannot be fully understood through the analysis of individual or contextual factors apart, but rather through their dynamic interaction. This is especially relevant in complex and high-pressure environments, such as advanced manufacturing. In such contexts, resilience emerges as a multilayered construct, where personal cognitive-emotional resources and organizational or social support systems are deeply intertwined. As highlighted in recent research [20], considering both dimensions simultaneously allows for a more accurate understanding of how workers adapt and thrive amidst organizational change, technological disruption, and operational stress.

In line with this perspective, in this study, psychological resilience is conceptualized as a multidimensional and context-sensitive capacity that enables workers to cope with stress, adapt to changes related to technological and organizational demands, and maintain effective functioning in complex and evolving manufacturing environments [20]. More specifically, resilience is framed as a higher-order construct encompassing 2 interrelated domains: individual resilience (eg, self-efficacy, cognitive flexibility, and change adaptation) and contextual resilience (eg, social support and organizational resilience). These dimensions jointly influence workers’ capacity to adapt to technological and organizational challenges in advanced manufacturing settings, forming an integrated conceptual model that underpins the development of the Change Adaptation Resilience Evaluation Questionnaire for Industry 5.0 (CARE-QI 5.0) [20]. These dimensions were selected as theoretically grounded components of resilience rather than independent constructs, in line with evidence highlighting the role of both individual resources and contextual protective factors in influencing adaptive functioning under stress [21,27,28]. Given these distinctions, work-related resilience can be broadly considered as the capacity to cope with and overcome adversity in occupational settings, emerging stronger and more competent as a result [32]. While existing research has addressed both individual and environmental dimensions of resilience, there is currently no unified theoretical model specifically tailored to industrial work environments. Despite the growing interest in resilience within high-tech manufacturing settings, the literature lacks psychometrically validated tools designed to assess resilience in such contexts [20]. To date, only Babamiri [33] has developed a tool related to HRI. However, it comprises only 3 items and fails to capture resilience as a multidimensional construct. In light of these limitations, this study introduces a new psychometric instrument: CARE-QI 5.0, specifically developed to assess and conceptualize psychological resilience in advanced manufacturing environments aligned with the principles of Industry 5.0.

### Aims

The overarching aim of this study was to develop and conduct an initial evaluation of the CARE-QI 5.0, a new multidimensional self-report instrument designed to assess psychological resilience among industrial workers in high-tech manufacturing environments. In line with the human-centric principles of Industry 5.0, this study pursued the following objectives:

- To conceptualize and operationalize psychological resilience in technologically advanced manufacturing contexts by integrating personal and contextual protective factors derived from existing theoretical and empirical frameworks.
- To develop and refine a context-sensitive set of items through a mixed methods approach that included expert review and focus group feedback, thereby enhancing the semantic clarity and contextual relevance of the instrument.
- To pilot the revised questionnaire in real-world high-tech industrial settings, evaluating its preliminary psychometric properties, including internal consistency, interitem correlations, and subscale coherence.

## Methods

### Participant Recruitment

Participants were recruited from 2 distinct manufacturing companies located in Northeastern Italy, both operating in technologically advanced production sectors. One company specializes in the design and production of high-precision mechanical components primarily for the pharmaceutical and packaging industries, while the other is a leading provider of flexible packaging solutions, focusing on innovation, sustainability, and advanced printing technologies for food and consumer goods.

For the focus group phase, inclusion criteria were established to ensure representativeness and alignment with the study’s aims. Specifically, participants were required to (1) be operators in high-tech companies, (2) ensure a balanced distribution by gender and age, and (3) be fluent in Italian to guarantee full comprehension and effective participation in the qualitative discussions.

For the pilot testing phase, a separate group of workers was recruited. Inclusion criteria for this phase included being operators employed in high-tech manufacturing companies and being fluent in Italian.

It is important to note that the participants involved in the focus groups differed from those who later took part in the pilot testing phase. This methodological separation was implemented to ensure that insights gathered during the qualitative development of the questionnaire did not bias its subsequent evaluation during pilot administration [34].

### Ethical Considerations

All participants were recruited voluntarily through internal company communications (eg, human resources announcements and mailing lists) as well as via digital and paper flyers specifically created for the purposes of this study. Informed consent was obtained from all participants electronically at the beginning of the digital questionnaire, prior to their involvement. No compensation was provided to participants. Data were collected and stored in compliance with applicable privacy regulations, ensuring the confidentiality and anonymity of participants’ data. Data collection procedures complied with the ethical standards outlined in the Declaration of Helsinki [35] and received 2 separate approvals from the Ethics Committee of the University of Padova (protocol 871-c, 2025 for the focus group sessions and 991-b, 2025 for the pilot administration).

### Design

**Figure 1.**
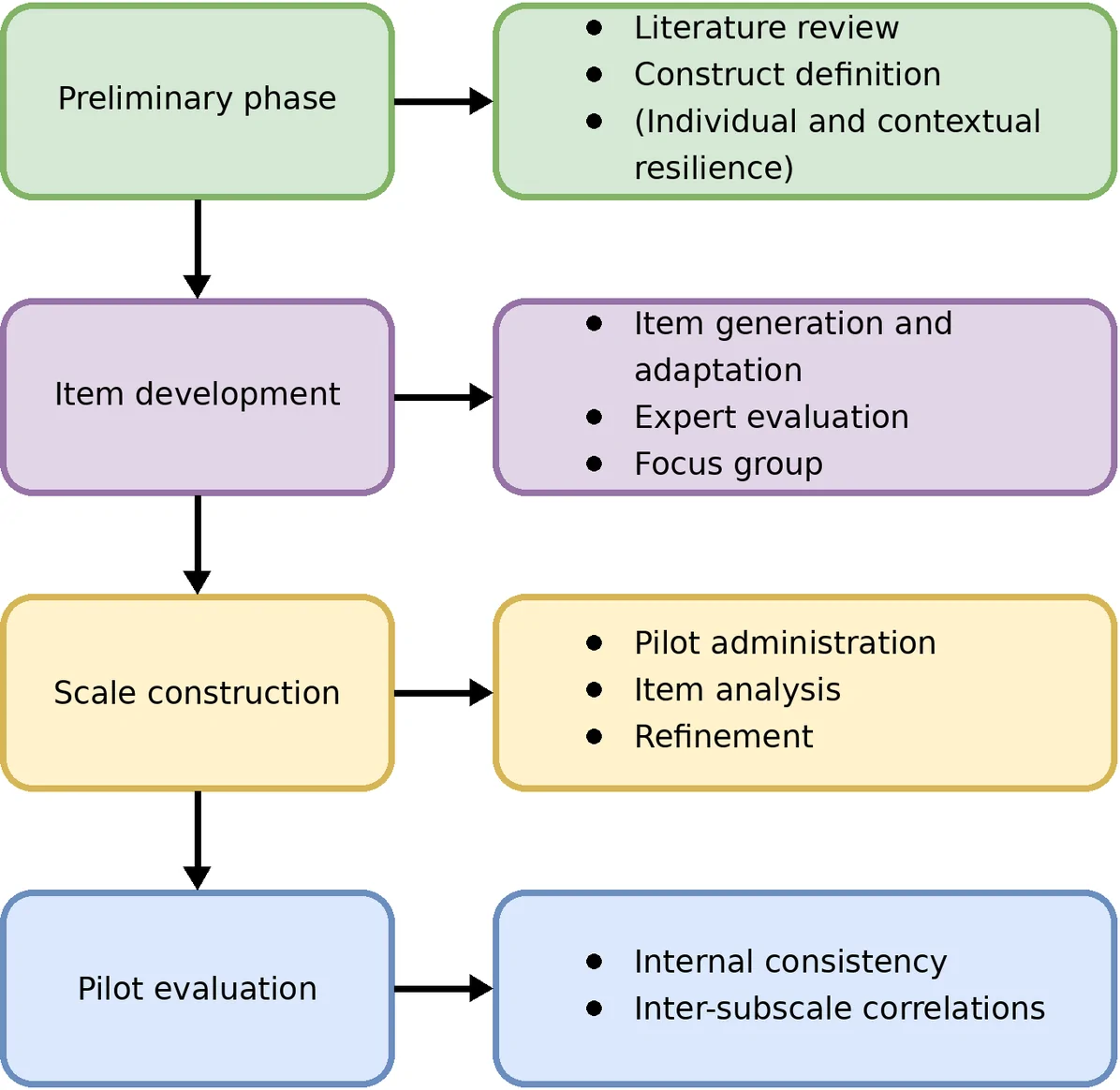
Development process of the CARE-QI 5.0 following best-practice guidelines for scale development.

An exploratory sequential mixed methods approach was used for the development of the CARE-QI 5.0 questionnaire, following established best-practice guidelines and scale development and validation frameworks [34,36-38]. This comprised a structured, multiphase process encompassing construct definition, item generation, expert evaluation, target population feedback, and pilot psychometric evaluation. The item development process followed an iterative refinement logic, in which they were not strictly retained or excluded based on a single quantitative criterion but were progressively revised, reworded, or replaced across phases, integrating expert judgment and feedback from the target population [34,36-38]. In line with these guidelines, the overall procedure is summarized in Figure 1, comprising 4 key stages.

First, we conducted a comprehensive literature review across academic databases to identify core constructs and relevant existing instruments. Drawing on these results, we generated and contextually adapted questionnaire items from validated sources. Next, the initial version of the instrument underwent expert review to assess content validity. Third, following item refinement, 2 separate focus groups, lasting 60 minutes each, were conducted with workers from 2 different manufacturing companies to obtain qualitative feedback on item clarity and comprehensibility. Finally, pilot administration of the revised digital questionnaire was conducted as part of a case study, allowing for initial preliminary evaluation aimed at exploring the psychometric properties of the instrument in a real-world industrial context. The digital survey also collected information on participants’ sociodemographic, occupational, and technology-related characteristics. Specifically, data were gathered on sex, age, job tenure, job role, work tasks, type of contract, technology readiness level (basic, intermediate, and high), frequency of technology use from 1 (“never”) to 5 (“always”), years of experience with such technologies, and whether they had received any related training. The duration of the compilation was approximately 25 minutes. The 4 major phases are specifically described step by step.

### Literature Review for Instrument Development

A deductive strategy for the selection of main constructs was applied, following established methodological guidelines [38]. This process involved a comprehensive review of existing theoretical models and empirical literature through Scopus, Web of Science, PubMed, and Google Scholar. This was followed by a series of iterative brainstorming sessions among the authors to identify the most relevant dimensions of psychological resilience. The selection of main constructs for the questionnaire aimed to provide a comprehensive and representative theoretical structure of worker resilience within advanced manufacturing contexts, in line with Industry 5.0 principles. Items were selected and adapted from validated instruments to ensure theoretical coherence and psychometric robustness, rather than being developed ex novo [34,36,38]. The selection of constructs and items followed a theory-driven approach, conceptualizing them as interrelated components of resilience rather than independent measures. In this framework, resilience was defined as a multidimensional construct influenced by the interaction between individual resources and contextual factors in technologically evolving work environments [20]. Accordingly, each dimension of the questionnaire was derived from established psychometric tools commonly used in psychological research [34-39]. Preference was given to tools that have been validated on Italian populations or adapted to the Italian sociocultural context by reporting good or excellent internal consistency values (Cronbach α≥0.70) in their validated Italian versions. Items were viewed and selected through a comparative process among the authors of this study. During this phase, items that were most representative of each specific subdimensional construct within the broader model were selected. For each construct, priority was given to items that demonstrated both theoretical clarity and contextual relevance. Subsequently, the authors proposed a context-specific readaptation of the selected items, in line with high-tech manufacturing environments.

### Experts’ Evaluation: Item Clarity Assessment

The first version of the CARE-QI 5.0 questionnaire was assessed by 3 experts in work and organizational psychology as well as HRI, who independently gave feedback regarding the clarity and comprehensibility of each item, without being informed of the questionnaire’s underlying factor structure. The assessors rated each item dichotomously (yes or no), reporting if it was clear or unclear by further providing qualitative comments to suggest any changes. Experts also provided open-ended comments to improve the semantic alignment of the items with the target population.

### Two Focus Groups

Two focus group sessions were conducted within 2 manufacturing companies known for their high-tech machinery usage, by implementing the guidelines described by Freeman [39]. This inductive approach, which involves collecting opinions from the target population to corroborate item clarity and their contextual relevance, is recognized as a pivotal step for the questionnaire development and validation process [40,41]. The facilitator (GB) read each item aloud while the text was projected on a monitor, and 2 observers (AV and ST) took notes of the participants’ nonverbal expression. The questionnaire was implemented on the *Qualtrics* platform (Qualtrics International Inc). Participants were asked to indicate whether the item was clear, unclear, or ambiguous by responding “yes” or “no” accordingly. This phase was inspired by cognitive interviewing [42], integrating a hetero-directed evaluation. The evaluation primarily focused on participants’ judgments of item clarity, but it was also enriched by their spontaneous comments. Although no formal probing scripts or structured think-aloud protocols were used, participants frequently shared their interpretations, suggested alternative wordings, and pointed out ambiguities. These qualitative insights offered valuable information about comprehension processes. All observations were audio recorded and transcribed verbatim to identify potential sources of misunderstanding and to refine the wording of the questionnaire items.

### The Pilot Administration

Following the item pool refinement, the CARE-QI 5.0 was administered through a pilot session to the same abovementioned recruited companies. The main purpose of this validation step concerned the examination of the internal validity of the questionnaire, which was filled out using the *Qualtrics* platform. Data were collected over a 2-week period. All items were scored on a 5-point Likert scale, ranging from 1 (“totally disagree”) to 5 (“totally agree”).

### Data Analysis

Statistical analyses were progressively conducted across each phase of the refinement process. As such, each version of the questionnaire integrated adjustments derived from the previous step, with the goal of enhancing item clarity and contextual relevance.

For the experts’ evaluation phase, item clarity and comprehensibility were assessed using an agreement index based on dichotomous judgments (clear or unclear) provided by 3 experts. For each item, we calculated the proportion of experts who rated it as clear. Items receiving full agreement (3/3 experts; 100% agreement) were retained without modification, whereas items with lower agreement were revised based on the experts’ qualitative feedback. Expert agreement was used to identify items that could be retained without modification, whereas items not reaching full agreement were further refined rather than discarded, in line with the iterative process [34,36,38].

For the focus groups, both quantitative and qualitative analyses were performed. Frequencies and percentages were computed for each item to identify potential comprehension issues, and content analysis was applied to summarize key themes from participants’ feedback.

In the pilot phase, all statistical analyses were performed using RStudio (Posit, PBC) [43]. Descriptive statistics were computed for each item and each subscale of the CARE-QI 5.0 questionnaire.

Internal consistency was assessed using Cronbach α and McDonald ω total coefficients, calculated separately for each subscale. For each α estimate, the corresponding 95% CI (lower and upper bounds) was also computed to evaluate the stability and precision of internal consistency estimates.

Pearson correlation coefficients (*r*) were calculated to assess interitem correlations within each subscale to evaluate internal coherence and identify potentially misfitting items. In addition, item-total correlations were examined to verify the contribution of each item to its respective subscale.

Furthermore, intersubscale Pearson correlations (*r*) were computed to explore the relationships among the identified dimensions of the questionnaire. This step aimed to preliminarily assess the internal structure and construct validity of the tool, consistent with the hypothesized hierarchical model of CARE-QI 5.0. Moreover, the subscales were correlated with age, job tenure, technology readiness level (basic, intermediate, and high), frequency of technology use, and years of experience with such technologies. The strength of interfactor relationships was interpreted based on Cohen’s [44] guidelines, where *r* values of 0.10, 0.30, and 0.50 are considered small, medium, and large effects, respectively. In addition, intersubscale correlations exceeding *r*>0.85 were interpreted as potential indicators of construct overlap, suggesting that the associated dimensions may be measuring substantially similar or redundant constructs [45].

## Results

### Results of the 4 Phases

**Figure 2.**
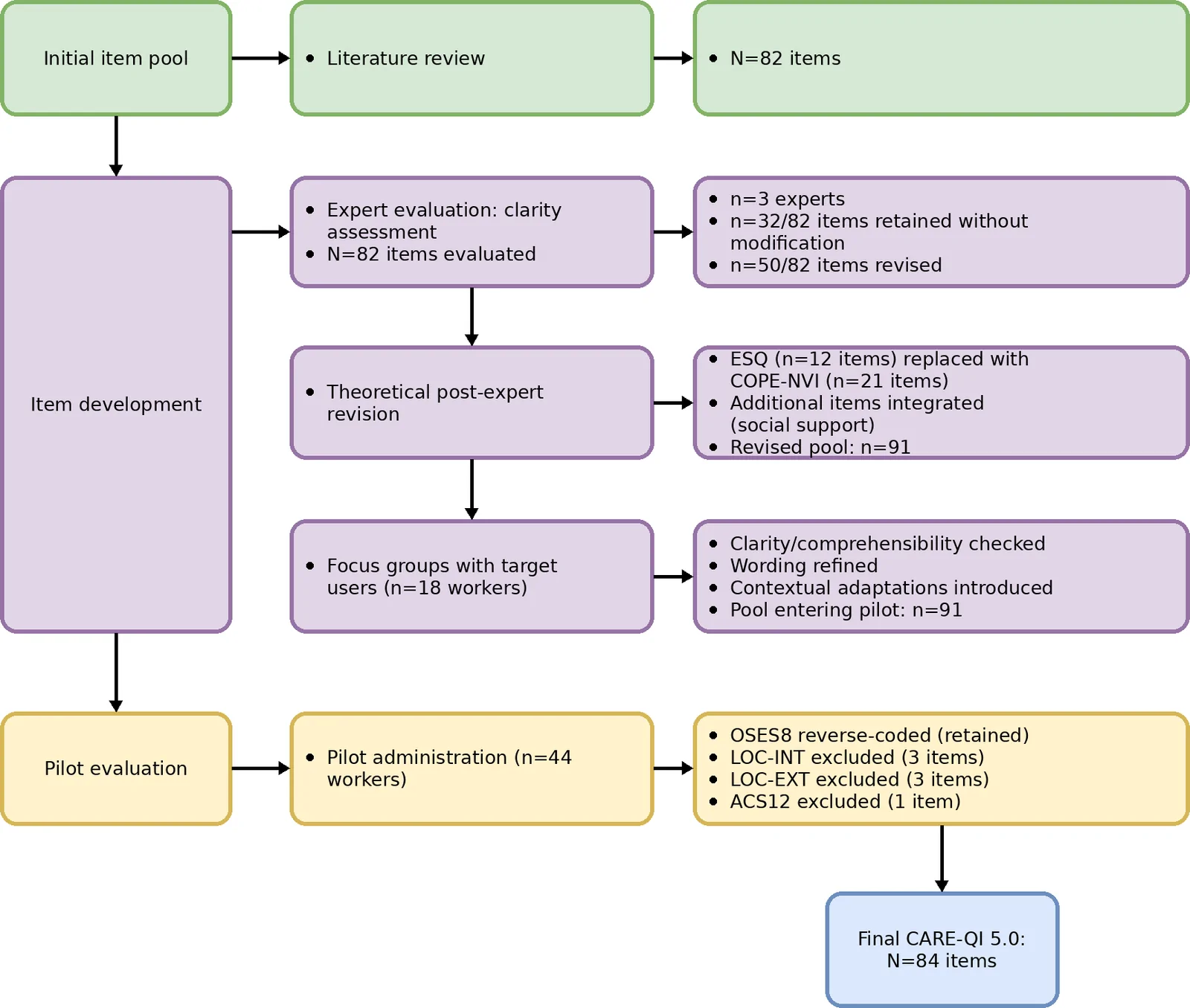
Flow diagram of the item selection and refinement process. ACS: Acceptance of Change Scale; CARE-QI 5.0: Change Adaptation Resilience Evaluation Questionnaire for Industry 5.0; COPE-NVI: Coping Orientation to Problems Experienced–Italian Version; ESQ: Emotional Style Questionnaire; LOC-EXT: external locus of control; LOC-INT: internal locus of control; OSES: Occupational Self-Efficacy Scale.

Across the different phases of the study, the CARE-QI 5.0 was developed by integrating and adapting items derived from multiple validated questionnaires, which were progressively refined throughout the process. The results of each development phase are summarized in Tables 1-7. Specifically, Table 1 reports the initial item pool derived from the literature review and existing instruments, Tables 2 and 3 present the results of the expert evaluation and subsequent refinement of the selected items, Tables 4 and 5 describe the characteristics of the pilot sample and the technologies used, Table 6 reports the reliability analyses, and Table 7 presents the final set of questionnaires and subscales included in the CARE-QI 5.0 for the pilot phase. Moreover, item selection and refinement followed an iterative process across the 4 study phases. As illustrated in Figure 2, the initial pool of 82 items was first evaluated by experts, resulting in a subset retained without modification (n=32), and the remaining items were revised based on qualitative feedback. Subsequently, theoretically driven modifications were introduced, including the replacement of the Emotional Style Questionnaire (ESQ; 12 items) with the Coping Orientation to the Problems Experienced (COPE-NVI; 21 items), leading to an expanded pool of 91 items. This version was then evaluated through focus groups, which informed further wording refinements and contextual adaptations. The final version administered in the pilot phase comprised 91 items.

**Table 1.** The first set of questionnaires used in the development of Change Adaptation Resilience Evaluation Questionnaire for Industry 5.0.

Construct	Tool	Cronbach α	Subscales selected
Self-efficacy	Occupational Self-Efficacy Scale (OSES) [46]	Overall α=0.69‐0.77	Problem solving (OSES-PS); and confidence to persevere in the face of difficulties (OSES-CD)
Emotion regulation strategies	Emotional Style Questionnaire (ESQ) [47] (excluded after expert evaluation)	Resilience: α=0.70; self-awareness: α=0.62; and sensitivity to context: α=0.71	Resilience (ESQ-RE); self-awareness (ESQ-SA); and sensitivity to context (ESQ-SC)
Locus of control	Locus of Control (LOC-L) [48]	Total scale: α=0.81	Internal locus of control (LOC-INT); and external locus of control (LOC-EXT)
Change management and adaptation	Acceptance of Change Scale (ACS) [49]	Predisposition to change: α=0.83; change seeking: α=0.80; and positive reaction to change: α=0.75	Predisposition to change (ACS-PTC); seeking to change (ACS-STC); and positive reaction to change (ACS-PRTC)
Cognitive flexibility	Multidimensional Psychological Flexibility Inventory (MPFI) [50]	Cognitive flexibility: acceptance: α=0.85; and committed action: α=0.88. Cognitive inflexibility: fusion: α=0.93; inaction: α=0.94	Cognitive flexibility (acceptance and committed action; MPFI-CF); and cognitive inflexibility (fusion and inaction; MPFI-CINF)
Social support	Multidimensional Scale of Perceived Social Support (MSPSS) [51]; Health and Safety Executive Stress Indicator Tool (SIT) [52]; and Survey of Perceived Organizational Support (SPOS) [53]	MSPSS: family: α=0.92; and friends: α=0.96. SIT: supervisor: α=0.85; and colleagues: α=0.86. SPOS: organization: α=0.81	Friends (SS-Friends); family (SS-Family); colleagues (SS-Coll); supervisors/managers (SS-S/M); and organization (SS-ORG)
Organizational resilience	Self-Evaluation of Resilience (SEOR) [54]	Skill-based resilience: α=0.90; and individual-based resilience: α=0.81	Skill-based resilience (SEOR-SR); and individual-based resilience (SEOR-IR)

**Table 2.** Proportion of expert agreement on item clarity.

Questionnaires	Total items, n	Items with full agreement (3/3 experts), n	Percentage with full agreement (%)
Total	82	32	39
OSES^a^	10	6	60
ESQ^b^	12	1	8
LOC-L^c^	6	3	50
ACS^d^	12	3	25
SS^e^	13	11	85
MPFI^f^	20	5	25
SEOR^g^	9	3	33

a OSES: Occupational Self-Efficacy Scale.

b ESQ: Emotional Style Questionnaire.

c LOC-L: Locus of Control.

d ACS: Acceptance of Change Scale.

e SS: Social Support.

f MPFI: Cognitive Flexibility.

g SEOR: Self-Evaluation of Resilience.

**Table 3.** The second set of questionnaires used in the development of Change Adaptation Resilience Evaluation Questionnaire for Industry 5.0.

Construct	Tool	Cronbach α	Subscales selected
Self-efficacy	Occupational Self-Efficacy Scale (OSES) [46]	Overall α=0.69‐0.77	Problem solving (OSES-PS); and confidence to persevere in the face of difficulties (OSES-CD)
Coping strategies	Coping Orientation to Problems Experienced–Italian Version (COPE-NVI) [55], included after expert evaluation	Avoidance strategies: α=0.68; positive attitude: α=0.74; problem-oriented coping: α=0.77; and seeking social support: α=0.81	Avoidance strategies (COPE-AV); positive attitude (COPE-PA); problem-oriented coping (COPE-PO); and seeking social support (COPE-SSS)
Locus of control	Locus of Control (LOC-L) [48]	Total scale: α=0.81	Internal locus of control (LOC-INT); and external locus of control (LOC-EXT)
Change management and adaptation	Acceptance of Change Scale (ACS) [49]	Predisposition to change: α=0.83; seeking to change: α=0.80; and positive reaction to change: α=0.75	Predisposition to change (ACS-PTC); seeking to change (ACS-STC); and positive reaction to change (ACS-PRTC)
Cognitive flexibility	Multidimensional Psychological Flexibility Inventory (MPFI) [50]	Cognitive flexibility: acceptance: α=0.85; and committed action: α=0.88. Cognitive inflexibility: fusion: α=0.93; and inaction: α=0.94	Cognitive flexibility (acceptance and committed action; MPFI-CF); and cognitive inflexibility (fusion and inaction; MPFI-CINF)
Social support	Multidimensional Scale of Perceived Social Support (MSPSS) [51]; Health and Safety Executive Stress Indicator Tool (SIT) [52]; and Survey of Perceived Organizational Support (SPOS) [53]	MSPSS: family: α=0.92; friends: α=0.96. SIT: supervisor: α=0.85; colleagues: α=0.86. SPOS: organization: α=0.81	Friends (SS-Friends); family (SS-Family); colleagues (SS-Coll); supervisors/managers (SS-S/M); and organization (SS-ORG)
Organizational resilience	Self-Evaluation of Resilience (SEOR) [54]	Skill-based resilience: α=0.90; and individual-based resilience: α=0.81	Skill-based resilience (SEOR-SR); and individual-based resilience (SEOR-IR)

**Table 4.** Sociodemographic, occupational-related, and technology-related characteristics (N=44).

Variable	Values
Sex, n (%)	
Male	29 (65.9)
Nationality, n (%)	
Italian	41 (93.2)
Age (y), mean (SD)	45.12 (11.74)
Job tenure (y), mean (SD)	18.42 (8.55)
Job role, n (%)	
Operators	40(90.9)
Technician	4 (9.1)
Task, n (%)	
Assembly	17 (38.6)
Picking	2 (4.5)
Welding	2 (4.5)
Product quality control	2 (4.5)
Type of contract, n (%)	
Open ended	33 (75)
Fixed term	4 (9.1)
Interim	4 (9.1)
Other (ie, employee leasing and work under an independent contractor agreement)	2 (4.5)
Part time	1 (2.3)
Technology readiness level, n (%)	
Base	12 (27.3)
Intermediate	28 (63.6)
High	4 (9.1)
Frequency of technology use for working, mean (SD)	3.93 (1.17)
Years of experience with technology for working, mean (SD)	15.63 (8.91)
Training course for using technologies for working (yes), n (%)	33 (75)

**Table 5.** Technologies used for working (in decreasing order).

Technologies	Description	Values, n (%)
Robotic assembly lines	Fully automated robotized assembly lines	16 (36.36)
Measurement instruments	Calipers, micrometers, etc	16 (36.36)
Pillar drills, drilling, and tapping machines	Includes pillar drills, tapping, and other similar tools	12 (27.27)
Semi-manual machining	Electric or manual hybrid workstations	12 (27.27)
Loading and unloading robots	Robots for handling between stations	11 (25.00)
Assembly tables	Fixed or smart tables for manual or semiautomatic assembly	9 (20.45)
Automated handling systems	Conveyors, shuttles, and trolleys	9 (20.45)
Testing machines	For functional or quality testing	9 (20.45)
Lathe	These lathes use a computer to control the machining of the workpiece, ensuring precision and repeatability	8 (18.18)
Screwing, gluing, and welding machines	Automated joining via screws, glue, or welding	8 (18.18)
Digital systems for quality	Classification, defect detection, etc	7 (15.91)
Industrial computers	Computers embedded in manufacturing lines	7 (15.91)
Tracking or traceability software	Batch or product traceability tools	7 (15.91)
Real-time monitoring systems	Real-time performance or quality monitoring	6 (13.64)
Milling machine	They remove material from a workpiece using software to guide the movement of the milling cutter	5 (11.36)
Automatic saws	Programmable saws for cutting profiles and bars	5 (11.36)
Bending machines	Controlled bending equipment (CNC^a^)	5 (11.36)
Other	Unspecified or miscellaneous tools	5 (11.36)
Hydraulic and electronic presses	Hydraulic presses for forming or punching	4 (9.09)
Pick-and-place systems	Robotic systems for part handling	4 (9.09)
Cobot (collaborative robots)	Collaborative robots working with humans	3 (6.82)
Gantry, scara, and delta robots	Industrial robots with special architectures	3 (6.82)
Automatic punching machines	Machines that perform punching operations on sheet metal, controlled by software to position and execute punches	2 (4.55)
Waterjet cutting	Plasma-based material cutting	2 (4.55)
Injection molding	Plastic forming by high-pressure injection	2 (4.55)
Programmable logic controllers	Programmable logic controllers for process automation	2 (4.55)
Manufacturing execution system	Manufacturing execution systems	2 (4.55)
Laser scanners and 3D optical measurement systems	Optical scanners for dimension control	2 (4.55)
Laser cutting	Software-guided laser material cutting	1 (2.27)
Automated guided vehicles and autonomous mobile robots	Autonomous material-handling vehicles	1 (2.27)
Computer-aided design and computer-aided manufacturing	Computer-aided design and manufacturing tools	1 (2.27)

a CNC: Computer Numerical Control.

**Table 6.** Cronbach α and McDonald ω_total for each factor.

Factors	α	95% CI lower	95% CI upper	ω_total	95% CI lower	95% CI upper
OSES-PS^a^	0.79	0.63	0.87	0.71	0.55	0.83
OSES-CD^b^	0.84	0.75	0.90	0.88	0.79	0.93
COPE-AV^c^	0.87	0.70	0.94	0.88	0.80	0.93
COPE-PA^d^	0.63	0.35	0.78	0.69	0.50	0.82
COPE-PO^e^	0.79	0.68	0.87	0.82	0.72	0.89
COPE-SSS^f^	0.78	0.59	0.88	0.74	0.60	0.84
*LOCL-INT^g^*	*-0.04*	*-0.85*	*0.42*	*0.23*	*0.00*	*0.52*
*LOCL-EXT^g^*	*0.60*	*0.29*	*0.76*	*0.71*	*0.53*	*0.84*
ACS-PTC^h^	0.83	0.66	0.92	0.83	0.73	0.90
ACS-STC^i^	0.65	0.33	0.81	0.67	0.48	0.81
ACS-PRTC^j^	0.67	0.25	0.84	0.68	0.49	0.82
SS-Friends^k^	0.79	0.48	0.93	0.79	0.60	0.90
SS-Family^l^	0.82	0.63	0.92	0.83	0.68	0.91
SS-S/M^m^	0.89	0.81	0.94	0.90	0.84	0.94
SS-Coll^n^	0.85	0.71	0.92	0.86	0.76	0.92
SS-ORG^o^	0.93	0.88	0.96	0.94	0.89	0.96
MPFI-CF^p^	0.76	0.58	0.86	*0.55*	0.32	0.74
MPFI-CINF^q^	0.92	0.85	0.95	0.92	0.87	0.95
SEOR-SR^r^	0.94	0.89	0.97	0.94	0.90	0.96
SEOR-IR^s^	0.79	0.61	0.88	0.80	0.66	0.89
Total score	0.95	0.91	0.97	0.90	0.82	0.95
Individual resilience	0.93	0.79	0.97	0.84	0.74	0.91
Contextual resilience	0.96	0.94	0.98	0.96	0.93	0.98

a OSES-PS: Occupational Self-Efficacy Scale-Problem Solving.

b OSES-CD: Occupational Self-Efficacy Scale-Confidence to persevere in the face of difficulties.

c COPE-AV: Coping Orientation to Problems Experienced-Avoidance Strategies.

d COPE-PA: Coping Orientation to Problems Experienced-Positive Strategies.

e COPE-PO: Coping Orientation to Problems Experienced-Problem Oriented.

f COPE-SSS: Coping Orientation to Problems Experienced-Seeking Social Support.

g Excluded.

h ACS-PTC: Acceptance of Change Scale-predisposition to change.

i ACS-STC: Acceptance of Change Scale Seeking to Change.

j ACS-PRTC: Acceptance of Change Scale Positive Reaction to Change.

k SS-Friends: Social Support-Friends.

l SS-Family: Social Support-Family.

m SS-S/M: Social Support-Supervisors/Managers.

n SS-Coll: Social Support-Colleagues.

o SS-ORG: Social Support-Organization.

p MPFI-CF: Multidimensional Psychological Flexibility Inventory cognitive flexibility.

q MPFI-CINF: Multidimensional Psychological Flexibility Inventory cognitive inflexibility.

r SEOR-SR: Self-Evaluation of Resilience-Skill-based resilience.

s SEOR-IR: Self-Evaluation of Resilience-Individual-based resilience.

**Table 7.** The third and final set of questionnaires used in the development of CARE-QI 5.0.

Construct	Tool	Cronbach α	Subscales selected
Self-efficacy	Occupational Self-Efficacy Scale (OSES) [46]	Overall α=0.69‐0.77	Problem solving (OSES-PS); and confidence to persevere in the face of difficulties (OSES-CD)
Coping strategies	Coping Orientation to Problems Experienced–Italian Version (COPE-NVI) [55], included after expert evaluation	Avoidance strategies: α=0.68; positive attitude: α=0.74; problem oriented: α=0.77; and seeking social support: α=0.81	Avoidance strategies (COPE-AV); positive attitude (COPE-PA); problem oriented (COPE-PO); and seeking social support (COPE-SSS)
Change management and adaptation	Acceptance of Change Scale (ACS) [49]	Predisposition to change: α=0.83; seeking to change: α*=*0.80; and positive reaction to change: α=0.75	Predisposition to change (ACS-PTC); seeking to change (ACS-STC); and positive reaction to change (ACS-PRTC)
Cognitive flexibility	Multidimensional Psychological Flexibility Inventory (MPFI) [50]	Cognitive flexibility: acceptance: α=0.85; and committed action: α=0.88. Cognitive inflexibility: fusion: α=0.93; and inaction: α=0.94	Cognitive flexibility (acceptance and committed action; MPFI-CF); and cognitive inflexibility (fusion and inaction; MPFI-CINF)
Social support	Multidimensional Scale of Perceived Social Support (MSPSS) [51]; Health and Safety Executive Stress Indicator Tool (SIT) [52]; and Survey of Perceived Organizational Support (SPOS) [53]	MSPSS: family: α=0.92; and friends: α=0.96. SIT: supervisor: α=0.85; and colleagues: α=0.86. SPOS: organization: α=0.81	Friends (SS-Friends); family (SS-Family); colleagues (SS-Coll); supervisors/managers (SS-S/M); and organization (SS-ORG)
Organizational resilience	Self-Evaluation of Resilience (SEOR) [54]	Skill-based resilience: α=0.90; and individual-based resilience: α=0.81	Skill-based resilience (SEOR-SR); and individual-based resilience (SEOR-IR)

### Literature Review

A comprehensive literature review led to the identification of a set of individual and contextual constructs empirically associated with resilience in organizational settings. Personal factors refer to internal dispositions and cognitive-emotional resources that individuals mobilize when facing adversity, such as self-efficacy, locus of control, emotion regulation strategies, and cognitive flexibility [24-30]. Conversely, contextual factors pertain to environmental resources and systemic support mechanisms, such as perceived social support and organizational adaptability, that fall outside the individual’s direct control but critically shape their capacity for resilience [29,31].

Accordingly, the CARE-QI 5.0 scale was developed by organizing these constructs into 2 higher-order dimensions under the overarching factor of resilience: (1) personal factors, comprising the subdimensions of self-efficacy, locus of control (internal and external), emotion regulation strategies, change management and adaptation, and cognitive flexibility; and (2) contextual factors, comprising social support and organizational resilience. This theoretical model informed the development of an initial item pool consisting of 82 items, all distributed across the identified subdimensions. The selection of the underlying measurement tools for each construct was based on their conceptual alignment with the target population (ie, industrial workers), empirical validity, psychometric soundness, and adaptability to high-tech occupational contexts. Table 1 summarizes the constructs, the original instruments, Cronbach α values (all >0.70), and the first set of the selected subscales for the development of CARE-QI 5.0.

In particular, for the cognitive flexibility dimension, items were adapted from the Multidimensional Psychological Flexibility Inventory (MPFI) [50], which originally includes 6 first-order factors loading onto two higher-order constructs: flexibility and inflexibility. In this study, only these two broader domains were retained, omitting the subfactors, to provide a more parsimonious and context-sensitive model tailored to high-tech manufacturing environments.

Furthermore, the Social Support (SS) dimension was constructed by integrating items from three validated instruments to represent distinct sources of support relevant to workplace dynamics: (1) 4 items were drawn from the Italian version of the Multidimensional Scale of Perceived Social Support [51], assessing support from family and friends; (2) 6 items from the Health and Safety Executive Stress Indicator Tool (SIT) [52] measured support from colleagues and supervisors or managers; and (3) 3 items from the Survey of Perceived Organizational Support [53] addressed support from the organization. This integrated approach allowed for a multidimensional and occupationally relevant operationalization of perceived social support.

### Experts’ Evaluation: Item Clarity Assessment

As summarized in Table 2, expert judgments were used to assess item clarity and comprehensibility. Of the 82 items originally included across 7 questionnaires, 32 items (39%) achieved full expert agreement on clarity. Notably, the SS questionnaire showed the highest agreement, with 85% of its items (11/13) rated as fully clear. In contrast, the ESQ [47] presented significant issues, with only 1 (8%) of 12 items receiving unanimous agreement. Experts provided extensive qualitative feedback regarding the ESQ’s lack of contextual relevance and overly complex phrasing, especially in relation to workplace dynamics. As a result, the ESQ was removed from the battery and replaced with the COPE-NVI [55] (21 items), which better aligns with the aim of assessing emotional regulation strategies within occupational contexts and demonstrates solid internal consistency [55]. These revisions, theoretically driven and supported by experts’ feedback, were made prior to pilot testing and are reflected in the final pool of 91 items. Table 3 reports the second set of questionnaires used in the development of CARE-QI 5.0.

### Focus Groups

#### Participants of the Focus Groups

A total of 18 operators participated in 2 focus groups (mean age 49.22, SD 9.95 y). The sample was evenly split by sex (50% male and 50% female). Participants had an average job tenure of 29.53 (SD 12.04) years, with a range from 1 to 37 years. Most participants were Italian (n=13, 72.2%), while the remaining 5 (27.8%) were foreign nationals fluent in Italian.

The first focus group consisted of 8 participants (men: n=7, 87.5%), with a mean age of 45.9 (SD 12.12) years. The second group included 10 participants (women: n=8, 80%), with a mean age of 51.9 (SD 6.63) years.

#### Results Emerged From the Two Focus Groups

The overall clarity assessment indicated that 90.7% of the items were rated as clear by participants, while only 9.3% were identified as unclear. Suggestions for improvement primarily concerned wording adjustments to better reflect the manufacturing context. In particular, participants recommended avoiding the use of anglicisms (eg, *routine* and *feedback*) and suggested replacing generic terms such as “organization” with “company” to increase contextual relevance. Specific concerns emerged regarding the MPFI [50], whose items were perceived as ambiguous in distinguishing between work-related and personal domains. To address this, all MPFI items were reformulated to remove references to the workplace, ensuring they referred solely to individual characteristics across contexts.

### Pilot Administration

#### Participants of the Pilot Study: Sociodemographic and Occupational-Related Characteristics

Table 4 presents the sample’s sociodemographic and occupational-related characteristics.

A total of 44 participants completed the revised version of the questionnaire as part of the pilot administration. Age data were available for 43 (97.7%) participants (mean age 45.12, SD 11.74 y). No participants were excluded from the analyses. Most participants were Italian men (n*=*29*,* 65.9%) and had a mean job tenure of 18.42 (SD 8.55) years. The majority worked as operators (n*=*40, 90.9%), *while technicians (*n=4, 9.1%) were less represented. Assembly was the most common task (n=17, 38.6%), followed by welding (n=2, 4.5%), picking (n*=*2*,* 4.5%), and product quality control (n*=*2, 4.5%). Most participants had an open-ended contract (n*=*33, 75%), with fewer on interim (n=4, 9.1%), fixed-term contract (n=4, 9.1%), part-time contracts (n=1, 2.3%), or other types of contract (n=2, 4.5%), such as employee leasing and work under an independent contractor agreement.

#### Technology Characteristics

As reported in Table 4, regarding technology readiness, 63.6% reported an intermediate level, 27.3% reported a basic level, and only 9.1% reported a high level. On average, participants reported using technology frequently in their work (mean 3.93, SD 1.17) and had been working with such technologies for an average of 15.63 (SD 8.91) years. The majority had received training (n=33, 75%) for using such technologies within their workplace. Moreover, Table 5 presents the technology used for working. Among the wide range of technologies reported by participants, the most frequently used were robotic assembly lines (n=16, 36.36%) and measurement instruments for calibration and testing (n=16, 36.36%), followed by semimanual machining stations (n=12, 27.27%) and pillar drills or similar drilling or tapping machines (n=12, 27.27%). Other commonly mentioned technologies included loading and unloading robots (n=11, 25.00%), assembly tables (n=9, 20.45%), testing machines (n=9, 20.45%), and automated material handling systems such as conveyors and trolleys (n=9, 20.45%).

### Reliability of the CARE-QI 5.0 Subscales

As presented in Table 6, most factors demonstrated acceptable to excellent reliability. Internal consistency was assessed using both Cronbach α and McDonald ω_total. Specifically, alpha coefficients ranged from 0.63 related to the Positive attitude subscale of the COPE-NVI scale (COPE-PA) [55] to 0.94 related to the Skill-based Resilience of the Self-Evaluation of Resilience scale (SEOR-SR) subscale, with CIs generally supporting the stability of internal consistency.

However, the internal (LOC-INT) and external (LOC-EXT) locus of control subscales yielded suboptimal reliability estimates. In particular, the analysis of LOC-INT revealed a negative α coefficient (α=−.04, 95% CI −0.845 to 0.420), suggesting problematic item intercorrelations. LOCL3 (*“A well-prepared person always finds a satisfying job”*) showed a negative correlation with LOCL1 (*r*=−0.27) and a very weak correlation with LOCL2 (*r*=0.05). When LOCL3 was removed, internal consistency improved (α=.48) but still did not reach an acceptable threshold. Moreover, the LOC-EXT showed a low reliability (α=.595, 95% CI 0.287 to 0.759). On the basis of both statistical evidence and conceptual considerations, specifically, the deterministic and potentially externally oriented nature of LOCL3’s content, which aligns more with Levenson’s [56] depiction of external reinforcement expectancies than with Rotter’s [57] original definition of internal locus of control, both the LOCL-INT and LOCL-EXT subscales were excluded from the final version of the resilience scale. A similar issue emerged in the “Confidence to persevere in the face of difficulties” (α=.087, 95% CI −0.452 to 0.309) subscale (OSES-CD) of the Occupational Self-Efficacy Scale (OSES) [46]. Correlational analysis revealed that item OSES8 (*“When unexpected problems happen at work, I don’t handle them very well”*) was negatively associated with all other items in the factor (eg, OSES8-OSES7, *r*=−0.15; OSES8-OSES9, *r*=−0.33), suggesting an inconsistency in directionality. Although the item was conceptually intended to be reverse scored, this transformation had not been applied prior to the initial analysis. After correcting this and reverse-coding OSES8 of the OSES, the internal consistency of the OSES-CD subscale improved substantially (α=.84), supporting its inclusion in the final structure of the scale. Moreover, the MPFI-CF showed a lower ω value compared to α, suggesting that the scale’s internal consistency may be partially influenced by its multidimensional structure (acceptance and committed action as distinct but related facets). Despite this, the scale was retained due to its theoretical centrality in resilience models [58]. The global CARE-QI 5.0 score showed excellent reliability (α=.95; *ω*=0.90). At the domain level, both dimensions demonstrated good to excellent internal consistency. Specifically, both individual and contextual resilience showed excellent reliability (α=.93; *ω*=0.84; and α=.96; *ω*=0.96, respectively).

The final pool items were 85. Table 7 presents the third and final set of questionnaires used in the development of CARE-QI 5.0.

To further enhance the transparency of item-level functioning, a supplementary table (Table S4, Multimedia Appendix 1) was included reporting corrected item-total correlations and the change in internal consistency coefficients (Cronbach α and McDonald ω) if each item were deleted. This information provides a detailed overview of the contribution of each item to the internal consistency of its respective subscale.

#### Description and Contextual Relevance of CARE-QI 5.0 Subscales

##### Overview

To enhance interpretability within Industry 5.0 contexts, each subscale is briefly defined in relation to workers’ adaptive functioning in technologically advanced environments [12]. The full item catalog (Italian and English versions), together with their original sources and type of adaptation, is reported in Table S1 in Multimedia Appendix 2. All items were derived from validated instruments and adapted following a theory-driven approach [34,36-38]. Adaptations varied depending on the source scale and included contextual modifications to the work environment (eg, for ACS and COPE), minor linguistic and semantic refinements (eg, for OSES and SEOR), and temporal reframing from past to present to ensure consistency in item format (eg, for MPFI) while preserving the original construct content.

##### Self-Efficacy (OSES-PS and OSES-CD)

The self-efficacy subscales reflect workers’ perceived capability to manage complex and unpredictable work demands [59]. In Industry 5.0 environments, characterized by increasing HRI and system variability, *problem-solving self-efficacy* supports rapid decision-making and operational continuity, whereas *confidence in persevering* reflects persistence under prolonged uncertainty and disruptions [18,21,46].

##### Coping Strategies (COPE-AV, COPE-PA, COPE-PO, and COPE-SSS)

The coping strategy subscales represent behavioral and cognitive responses to stressors [60]. In high-tech manufacturing contexts, *problem-oriented coping* is particularly relevant for actively managing technological challenges and workflow disruptions, whereas *avoidance and positive attitude* may reflect regulatory processes that vary in adaptiveness depending on situational demands [61,62]. Seeking social support is critical in collaborative environments where problem-solving relies on interpersonal coordination [31].

##### Change Management and Adaptation (ACS-PTC, ACS-STC, and ACS-PRTC)

These dimensions capture workers’ orientation toward change, a core requirement in Industry 5.0 systems characterized by continuous technological evolution [12]. For example, *openness and proactive engagement with change* have been associated with better adjustment to organizational transformation and innovation [24].

##### Cognitive Flexibility (MPFI-CF and MPFI-CINF)

*Cognitive flexibility* is a central resilience mechanism that enables individuals to adapt to changing demands and regulate emotional responses under stress [25,27]. In contrast, *cognitive inflexibility* reflects rigid cognitive patterns that may hinder adaptation, particularly in complex and dynamic technological environments [25,27].

##### Social Support (SS Subdomains)

The social support subdomains represent a key contextual resource for resilience, influencing both emotional regulation and adaptive functioning [28,32,51-53]. In Industry 5.0 environments, where teamwork and human-technology collaboration are central, support from colleagues, supervisors, and organizations facilitates trust and fluency [63].

##### Organizational Resilience (SEOR-SR and SEOR-IR)

The organizational resilience subscales refer to the capacity of organizations to adapt and sustain functioning under disruption events [11]. In Industry 5.0, this includes investment in training, skill development, and adaptive work structures that support effective human-technology integration and collective problem-solving [14,54].

### Descriptives of the Items and Subscales

Table S2 in Multimedia Appendix 3 reports the descriptive statistics (mean, SD, skewness, and kurtosis) for each item and corresponding subscale of the newly developed CARE-QI 5.0 resilience questionnaire.

Overall, item means ranged from 1.80 to 4.25, suggesting a moderate-to-high endorsement of most items. Several items exhibited moderate negative skewness, indicating a tendency toward higher agreement levels. For example, OSES2 (“When I face difficulties at work, I remain calm because I can rely on my abilities”) and item SS7 from the supervisors/managers subscale (*“*My direct supervisor provides what I need to perform my job effectively*”*) showed pronounced negative skewness (−1.46 and −1.12, respectively), suggesting a potential ceiling effect. Some items also demonstrated leptokurtic distributions, such as OSES2 (kurtosis=5.47), indicating clustered responses around the mean.

At the subscale level, total means ranged from 1.94 (COPE-AV) to 3.94 (COPE-PO), highlighting notable variability in the endorsement of different resilience dimensions. Specifically, higher means were observed for problem-solving self-efficacy (OSES-PS=3.92), problem-oriented coping (COPE-PO=3.94), and cognitive flexibility (MPFI-CF=3.71), whereas lower scores characterized avoidance strategies (COPE-AV=1.94) and confidence to persevere in the face of difficulties (OSES-CD=2.07).

### Interitem Correlations Within Subscales and Their Relationships With Total Scale Scores

Interitem correlation analyses within each subscale were conducted to evaluate the internal homogeneity of the items and are reported in Table S3 in Multimedia Appendix 4. Overall, most items showed moderate to strong correlations with one another within their respective subscales, supporting their coherence in measuring the same latent constructs.

However, for the Acceptance of Change Scale (ACS) [49], item ACS12 (“I am aware of the changes and modifications occurring in my workplace”) displayed weak correlations with the other items in the Positive Reaction to Change (ACS-PRTC) subscale. Its removal led to an increase in internal consistency (from α=.666 to .673), suggesting limited contribution to the underlying construct. On the basis of both statistical evidence and conceptual clarity, ACS12 was excluded from the final version.

Thus, the final items included in the CARE-QI 5.0 were 84.

### Interrelationships Between the CARE-QI 5.0 Subscales: Preliminary Evidence of Structural Coherence

**Figure 3.**
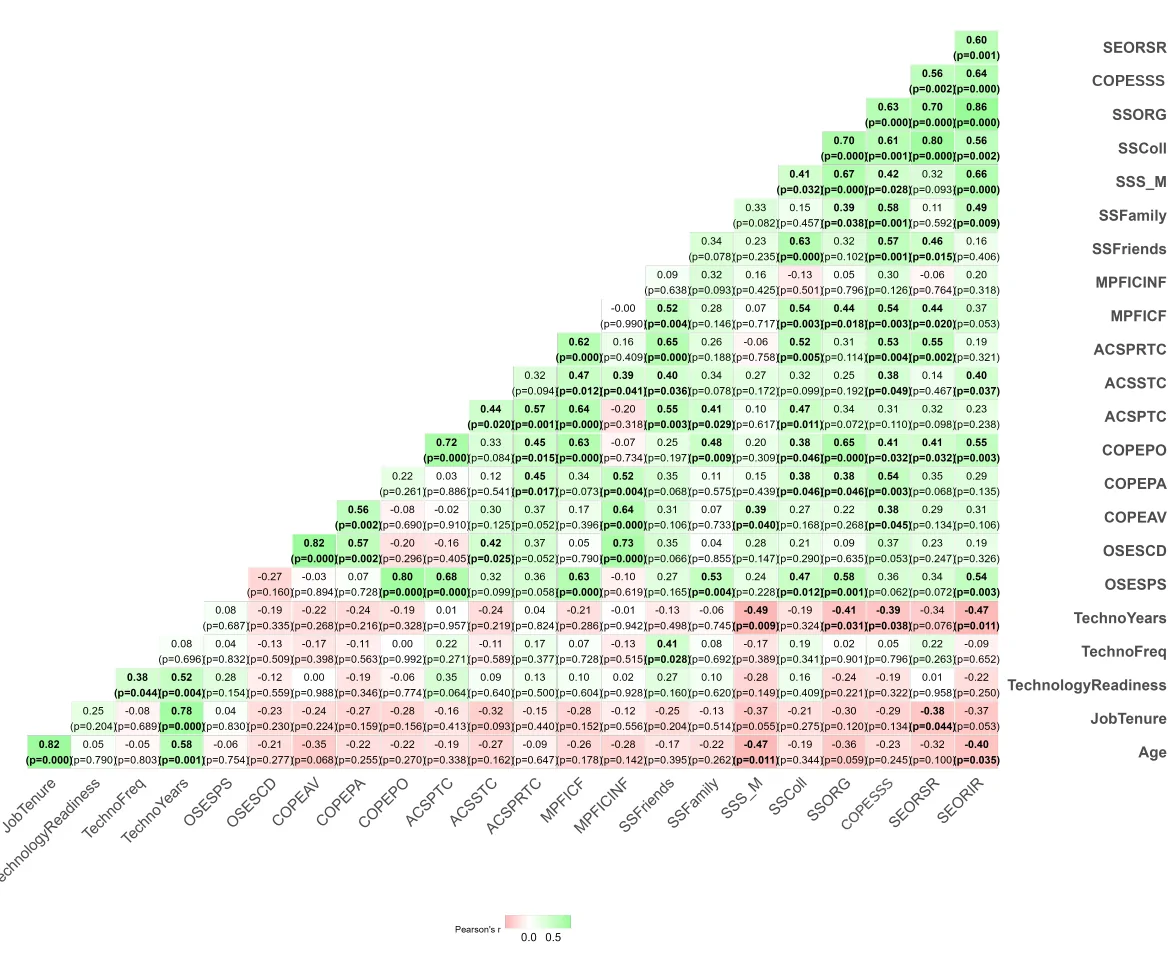
Correlations between the subscales of the Change Adaptation Resilience Evaluation Questionnaire for Industry 5.0. ACS-PRTC: positive reaction to change; ACS-PTC: predisposition to change; ACS-STC: seeking to change; COPE-AV: coping orientation to problems experienced-avoidance strategies; COPE-PA: coping orientation to problems experienced-positive strategies; COPE-PO: coping orientation to problems experienced-problem oriented; COPE-SSS: coping orientation to problems experienced-seeking social support; MPFI-CF: cognitive flexibility; MPFI-CINF: cognitive inflexibility; OSES-CD: Occupational Self-Efficacy Scale-confidence to persevere in the face of difficulties; OSES-PS: Occupational Self-Efficacy Scale-problem solving; SEOR-IR: Self-Evaluation of Resilience-individual-based resilience; SEOR-SR: Self-Evaluation of Resilience-skill-based resilience; SS-Coll: Social Support-colleagues; SS-Family: Social Support-family; SS-Friends: Social Support-friends; SS-ORG: Social Support-organization; SS-S/M: Social Support-supervisors/managers; TechnoFreq: frequency of technology use; TechnoYears: years of experience with technology.

As illustrated in Figure 3, problem-solving self-efficacy (OSES-PS) was positively associated with individual-based resilience (SEOR-IR), as well as with perceived support from the organization, colleagues, and family, cognitive flexibility (MPFI-CF), predisposition to change (ACS-PTC), and problem-oriented coping strategies (COPE-PO).

The dimension of perseverance in facing difficulties (OSES-CD) showed positive correlations with cognitive inflexibility (MPFI-CINF), seeking to change (ACS-STC), positive attitude (COPE-PA), and avoidance strategies (COPE-AV), indicating a less adaptive pattern of coping.

Both positive attitude and avoidance coping strategies were positively associated with cognitive inflexibility. Additionally, positive attitude was also positively correlated with support from the organization and colleagues and with positive reaction to change (ACS-PRTC); conversely, avoidance was positively associated with support from managers or supervisors.

Problem-oriented coping strategies (COPE-PO) were significantly and positively related to both dimensions of organizational resilience (SEOR-SR and SEOR-IR), support from the organization, colleagues, and family, cognitive flexibility, and 2 subdimensions of change management and adaptation (ie, ACS-PRTC and ACS-PTC).

The ACS-PTC subscale was positively correlated with support from colleagues, family, and friends and with cognitive flexibility. The ACS-STC dimension showed positive associations with seeking social support (COPE-SSS) and perceived support from friends, individual-based resilience, and both dimensions of cognitive (in)flexibility. The positive reaction to change dimension (ACS-PRTC) was positively associated with skill-based resilience (SS-Coll), support from colleagues and family, and cognitive flexibility.

Cognitive flexibility itself was positively related to skill-based resilience, as well as to COPE-SSS, the support from organization, colleagues, and friends, reinforcing its central role in adaptive functioning.

### Interrelationships Between the CARE-QI 5.0 Subscales and Occupational and Technology Variables

As shown in Figure 3, age was negatively associated with perceived social support from supervisors or managers and with individual-based resilience (SEOR-IR). In contrast, it showed positive correlations with both experience in using technology (TechnoYears) and job tenure. Job tenure was negatively correlated with skill-based resilience (SEOR-SR), while it was positively associated with technological experience. Technology readiness was positively related to both technology use frequency (TechnoFreq) and experience (TechnoYears). Moreover, technology use frequency was positively associated with support from friends. In addition, experience in using technology was negatively correlated with individual-based resilience, as well as with perceived support from supervisors or managers and COPE-SSS.

## Discussion

### Principal Findings

The overarching aim of this study was to develop and preliminarily evaluate the CARE-QI 5.0, a multidimensional self-report instrument designed to assess resilience in manufacturing environments. The scale is grounded in a global score of resilience, with a dual-factor model, that conceptualizes adaptability as the result of 2 interrelated components: individual resilience (eg, self-efficacy, coping strategies, cognitive flexibility, and change management and adaptation) and contextual resilience (eg, perceived social support and organizational resilience). This framework reflects the human-centered and sustainable vision of Industry 5.0, where the technological transformation of production systems must be accompanied by the promotion of workers’ well-being, agency, and psychological adaptability [12].

In particular, this human-centered shift involves increasing forms of HRI and HRC, which require operators to adapt not only to new tools but also to novel relational dynamics with intelligent technologies. Understanding how workers psychologically respond to such transitions is therefore crucial for the sustainable integration of advanced automation.

To ensure theoretical and contextual robustness, the development of the questionnaire followed a 4-phase mixed methods approach: (1) a literature review, (2) expert review, (3) focus groups with manufacturing workers, and (4) a pilot administration aimed at assessing the internal consistency and the correlational structure of the instrument.

### Insights From the Preliminary Evaluation of the CARE-QI 5.0

Building on the literature review, a panel of psychologists and HCI experts was involved in a structured content and semantic evaluation of the selected items. This expert review phase was instrumental in validating the proposed dual-factor model of resilience, encompassing both individual and contextual resources, and in defining the preliminary structure of the CARE-QI 5.0. Items that did not meet minimum thresholds for clarity, comprehensibility, or contextual appropriateness were modified or excluded. In particular, although the ESQ was theoretically consistent with core resilience dimensions [47], it was considered too abstract and insufficiently grounded in the lived experiences of industrial workers. Consequently, ESQ-derived items were replaced with items from the COPE-NVI [55], a tool offering a more concrete and behaviorally oriented operationalization of coping strategies and previously validated in occupational settings [55].

Participants generally confirmed the clarity of the items (with over 90% rated as “clear”) but also highlighted the presence of language that was either too abstract or unfamiliar, including anglicisms and psychologisms. On the basis of their suggestions, numerous items were reworded to improve operational clarity, and the wording of certain scales, such as cognitive flexibility, was adjusted to ensure their applicability to daily work tasks, interpersonal relations, and problem-solving in the manufacturing context. This process enhanced both the ecological validity and the practical usability of the CARE-QI 5.0 instrument in Industry 5.0 settings.

The pilot testing phase provided evidence of satisfactory internal consistency for the vast majority of subscales, with Cronbach α and McDonald ω coefficients ranging from acceptable to excellent. Notably, at the global level, the CARE-QI 5.0 demonstrated excellent reliability (α=.95 and ω=0.90), suggesting that the overall instrument shows strong internal consistency. At the domain level, both dimensions showed good to excellent reliability: individual resilience (α=.93; ω=0.84) and contextual resilience (α=.96; ω=0.96), further supporting the structural coherence of the instrument. Notably, the cognitive flexibility scale showed a lower ω value compared to alpha, which may reflect the multidimensional structure of the scale, as it comprises acceptance and committed action as theoretically distinct yet related aspects of psychological flexibility. Despite this, the scale was retained, given its theoretical centrality in resilience models: cognitive flexibility, intended as the ability to adapt one’s cognitive repertoire in response to changing demands, has been consistently identified as a core component of psychological resilience, particularly in technologically evolving work environments where continuous adaptation to new tools, processes, and roles is required [58]. Only the locus of control dimensions did not meet the reliability threshold and were therefore excluded from the final structure of the questionnaire, which now includes 84 items across the individual and contextual domains. Correlational analyses further supported the structural coherence of the instrument. All intersubscale correlations remained below the critical threshold of *r*=0.85, indicating an absence of multicollinearity and acceptable discriminant validity. On the basis of Cohen’s [44] guidelines, most associations between dimensions fell within the small to moderate range, suggesting related yet distinct constructs, consistent with a hierarchical model of resilience.

### Internal Structure and Construct Validity

To assess construct validity, correlations between resilience dimensions and sociodemographic variables were also examined. As expected, age was positively associated with both technological experience and job tenure; yet age was negatively associated with individual-based resilience and perceived support from supervisors or managers. Similarly, technological experience was negatively related to individual-based resilience and to seeking social support and perceived support from supervisors, suggesting that increased exposure to technology in the workplace may not necessarily be coupled with perceived psychosocial support or internal resilience resources. Conversely, technology readiness was positively associated with both technology use frequency and experience, and the frequency of technology use was positively related to support from friends, indicating that digital engagement may reflect more socially integrated or voluntary patterns of use.

From a psychological perspective, problem-solving self-efficacy emerged as a construct deeply embedded in adaptive functioning. Its positive associations with individual-based resilience, cognitive flexibility, support from key relational and organizational sources, predisposition to change, and problem-oriented coping strategies reinforce its role as a central competence underpinning resilient responses. This pattern reflects a flexible and agentic configuration, in which confidence in one’s problem-solving abilities coexists with openness to change and access to supportive contexts. In contrast, the perseverance in facing difficulties (OSESCD) showed a more complex and potentially maladaptive profile. While traditionally viewed as a marker of resilience, its positive associations with cognitive inflexibility, avoidance, positive attitude, and seeking to change, in the absence of links with organizational support or self-efficacy, suggest a form of rigid perseverance, more closely aligned with resignation or effortful persistence than with adaptive coping. This interpretation can be supported by the content of the OSESCD items (eg, “I avoid trying new things when they seem too difficult”), which may reflect passivity or learned helplessness, particularly under conditions of perceived threat or low external support.

A particularly nuanced picture emerged around avoidance and positive attitude strategies, which were both positively associated with cognitive inflexibility but also with cognitive flexibility. This dual alignment underscores their ambivalent nature: while they may serve immediate emotion-regulation functions, their adaptiveness likely depends on the broader cognitive-emotional configuration in which they operate [61]. When enacted within a flexible mindset and embedded in supportive environments, they may contribute to adjustment. However, when embedded in rigid cognitive structures, they risk becoming maladaptive coping habits, promoting disengagement or illusory optimism [62,64].

In line with this dynamic perspective, cognitive inflexibility was largely uncorrelated with external resources (eg, organizational resilience and social support) but was positively associated with rigid or passive profiles, including perseverance in facing difficulties, avoidance, and positive attitude. This reinforces its conceptualization as a marker of limited adaptability, associated with reduced access to both internal and external regulatory mechanisms.

Conversely, cognitive flexibility emerged as a central protective factor, positively linked to both skill- and individual-based resilience, seeking social support and perceived support from colleagues and the organization, and openness to change across all 3 change-related subdimensions. Its wide-ranging associations suggest a pivotal role in integrating coping strategies, relational support, and openness to innovation, especially in complex and evolving work environments, such as those characterizing advanced manufacturing. Problem-oriented coping strategies aligned consistently with cognitive flexibility, resilience dimensions, and social-organizational support, highlighting their embeddedness in broader adaptive networks rather than standing as isolated traits.

Taken together, these findings support a process-oriented view of resilience, which emerges not from single traits or isolated strategies but from the dynamic interplay of flexible cognition, effective coping, change orientation, and social-organizational resources. The value of any strategy appears to depend less on its nominal category and more on how it functions within broader patterns of adaptation and support.

### Technology Readiness and Implications for Psychological Resilience

In addition to the psychometric evaluation of CARE-QI 5.0, the pilot phase provided valuable insights into workers’ technological exposure, which is a critical contextual factor for interpreting resilience in Industry 5.0 environments [12]. Most participants reported an intermediate (63.6%) or basic (27.3%) level of technology readiness, with only a small subset (9.1%) indicating high readiness. Nevertheless, technology use in daily operations was generally frequent (mean 3.93, SD 1.17), and workers reported an average of over 15 years of experience (mean 15.63, SD 8.91) with advanced manufacturing technologies. The most commonly used tools included robotic assembly lines and precision measurement instruments (both reported by 36.36% of the sample), followed by semimanual machining stations and pillar drills (each 27.27%). A diverse array of technologies was also mentioned, including loading and unloading robots (25.00%), assembly tables, testing machines, and automated material handling systems (each 20.45%). Although less prevalent, the use of collaborative robots (6.82%) and AI-assisted systems, such as MES platforms and real-time monitoring tools, suggests an ongoing, albeit gradual, transition toward more integrated HRC scenarios. Notably, the majority of participants (n=33, 75%) had received specific training for using these technologies, highlighting the importance of organizational investment in upskilling as a facilitator of technological adaptation. This finding reinforces the conceptualization of resilience as an interaction between individual resources and contextual supports, thereby underscoring how technological competence, fostered through structured training, may serve as a protective factor in high-tech industrial settings. Correlational analyses offered further insight into the psychological significance of these technological variables. Technology readiness was positively associated with the frequency of technology use and years of technological experience.

### Practical Implications

The CARE-QI 5.0 seems to be a robust and innovative tool for assessing psychological resilience in organizational settings, especially those undergoing significant technological and structural changes. Developed within the theoretical framework of Industry 5.0, the instrument embraces a human-centered approach that acknowledges the complexity of workers’ experiences in increasingly automated and digitized environments.

CARE-QI 5.0 is further characterized by a multidimensional structure, conceptualizing resilience as a higher-order factor composed of 2 second-order domains: individual resilience and contextual resilience. These domains are in turn articulated through a series of first-order subdimensions, including self-efficacy, coping strategies, cognitive flexibility, change management and adaptation, perceived social support, and organizational resilience. This layered architecture enables a comprehensive yet nuanced evaluation of workers’ adaptive capacities, considering not only internal dispositions but also the quality of their social and organizational environment. Such a structure is particularly relevant in light of the challenges posed by Industry 5.0 transformations, which involve close interaction between human operators and advanced technologies, such as collaborative robots [20], real-time monitoring systems, and intelligent automation. These changes impact not only task demands and technical skills but also workers’ psychological adaptability, stress regulation, and relational dynamics. CARE-QI 5.0 can therefore serve as a valuable instrument for diagnosing resilience profiles and for monitoring how employees adjust to innovation over time. Its use may inform targeted interventions aimed at strengthening both personal and systemic protective resources. Clinical and work and organizational psychologists, HRC experts, and HR departments may use the tool to detect early signals of psychological difficulties, map areas of risk or resilience, and tailor actions to support workers’ well-being. Moreover, CARE-QI 5.0 enables organizations to move beyond generic wellness initiatives and adopt evidence-based strategies for promoting sustainable human performance. By identifying which dimensions of resilience are most challenged in a given context, or reduced change readiness, companies can enact specific and measurable improvements in their work environment and organizational culture.

Therefore, CARE-QI 5.0 provides not only a means of assessing workers’ adaptability but also a strategic foundation for building more resilient, inclusive, and future-ready workplaces in alignment with the social and technological principles of Industry 5.0.

### Limitations and Future Directions

Despite its promising contributions, this study presents several limitations that should be considered, also for future research. First, the psychometric evaluation of the CARE-QI 5.0 remains preliminary. While internal consistency and intersubscale correlations provided initial evidence of structural coherence, no factor-analytic validation was conducted. Given the scale’s multidimensional structure, with resilience conceptualized as a general construct composed of 2 higher-order domains (individual and contextual resilience) and multiple first-order subdimensions, future research should use exploratory factor analysis and confirmatory factor analysis, including second-order and bifactor models, to test the hierarchical configuration of the instrument. Second, the sample was small and drawn from a specific regional and sectoral context, namely, manufacturing companies located in Northeastern Italy, primarily within the mechanical and packaging sectors. While these sectors represent relevant use cases in Industry 5.0 transitions, the limited scope may restrict the generalizability of the findings. Further research is needed to assess the applicability and cultural fit of the instrument in other manufacturing domains among a large sample size. Third, an additional limitation concerns the expert review phase. The panel included a small sample size (n=3 experts), which limits the stability and interpretability of item-level agreement estimates. Moreover, the expert evaluation focused exclusively on item clarity and comprehensibility, without formally assessing item essentiality. As such, the current findings should be considered preliminary with respect to content validity. Notably, this limitation was partially mitigated by the inclusion of focus groups with target users, which provided complementary evidence on item comprehensibility and contextual appropriateness. Future research should involve a larger panel of subject matter experts (eg, 5‐10 or more) and adopt more robust content validity procedures, including the use of indices such as the Content Validity Index, or Aiken V, ideally accompanied by CIs. Future validation efforts should also assess multiple aspects of content validity, including relevance and comprehensiveness, potentially integrating both experts’ judgment and feedback representatives of the target population. Finally, although qualitative methods (eg, expert review and focus groups) contributed to the linguistic and contextual refinement of the items, no analysis of measurement invariance was conducted. To ensure that the CARE-QI 5.0 functions equivalently across key demographic groups (eg, gender, age, and professional role), future studies should assess its psychometric stability and fairness using multigroup confirmatory factor analysis.

### Conclusions

This study contributes to the growing effort to develop context-sensitive tools for assessing psychological resilience within the evolving landscape of contemporary manufacturing. Through a mixed methods approach with several processes, encompassing theoretical review, expert evaluation, focus groups, and pilot testing, the CARE-QI 5.0 was developed as a multidimensional instrument, grounded in a dual framework of individual and contextual resilience.

Preliminary findings support the internal consistency and conceptual distinctiveness of its subdimensions, revealing the dynamic interplay between personal coping resources, cognitive adaptability, and perceived social-organizational support in shaping workers’ capacity to respond to organizational and technological transitions. Correlational analyses suggest that constructs, such as problem-solving self-efficacy, cognitive flexibility, and support from supervisors and colleagues, form core adaptive clusters, while other patterns, such as rigid perseverance and avoidance strategies, highlight the risk of maladaptive coping when flexibility and support are lacking.

By capturing both individual dispositions (eg, self-efficacy and openness to change) and environmental supports (eg, social support and organizational culture), CARE-QI 5.0 offers a comprehensive and integrative framework for evaluating psychosocial adaptability in high-tech, human-centered contexts. This makes the instrument especially relevant to the principles of Industry 5.0, where technological innovation is increasingly intertwined with sustainability, inclusion, and well-being.

While further psychometric validation and cross-contextual applications are needed, CARE-QI 5.0 holds promise as both a research instrument and a practical tool for high-tech manufacturing environments. It can assist organizations in mapping resilience profiles, guiding the design of targeted interventions, and promoting psychosocial sustainability in technologically advanced workplaces. In doing so, it supports a vision of industrial innovation that places psychological adaptability and human value at the core of progress.

## Supplementary material

10.2196/83754 Multimedia Appendix 1 Item-level reliability diagnostic (corrected item-total correlations, Cronbach α, and McDonald ω if item deleted) for the CARE-QI 5.0 subscales.

10.2196/83754 Multimedia Appendix 2 CARE-QI 5.0 structure and items selected (N=84 items).

10.2196/83754 Multimedia Appendix 3 . Items’ descriptive statistics.

10.2196/83754 Multimedia Appendix 4 Interitem correlations within subscales with their total scales. *P<.05; **P<.001.

## Abbreviations

ACS: Acceptance of Change Scale
ACS-PRTC: positive reaction to change
ACS-PTC: predisposition to change
CARE-QI 5.0: Change Adaptation Resilience Evaluation Questionnaire for Industry 5.0
COPE: coping orientation to problems experienced
COPE-AV: coping orientation to problems experienced-avoidance strategies
COPE-PA: coping orientation to problems experienced-positive strategies
COPE-PO: coping orientation to problems experienced-problem oriented
COPE-SSS: coping orientation to problems experienced-seeking social support
ESQ: Emotional Style Questionnaire
HRC: human-robot collaboration
HRI: human-robot interaction
LOC-EXT: external locus of control
LOC-INT: internal locus of control
LOC-L: Locus of Control at Work
MPFI: Multidimensional Psychological Flexibility Inventory
MPFI-CF: cognitive flexibility
MPFI-CINF: cognitive inflexibility
MSPSS: multidimensional scale of perceived social support
OSES: Occupational Self-Efficacy Scale
OSES-CD: Occupational Self-Efficacy Scale-Confidence to persevere in the face of difficulties
OSES-PS: Occupational Self-Efficacy Scale-Problem Solving
SEOR-IR: Self Evaluation of Resilience-Individual-based resilience
SEOR-SR: Self Evaluation of Resilience-Skill-based resilience
SS: Social Support
