# Supplementary material for: Change Adaptation Resilience Evaluation Questionnaire for Industry 5.0 Among Workers in High-Tech Manufacturing Companies: Questionnaire Development and Pilot Evaluation Study
Source: JMIR Hum Factors. 2026 Aug 28;13:e83754. doi: 10.2196/83754 (PMC13524363; doi:10.2196/83754)
Supplement: Supplementary file 1 [file humanfactors-v13-e83754-s001.docx]

**Table S4.** Item-level reliability diagnostics (corrected item-total correlations, Cronbach’s alpha and McDonald’s omega if item deleted) for the CARE-QI 5.0 subscales

| **Subscale** | **Item** | **Corrected item-total correlation** | **Cronbach’s alpha if item deleted** | **McDonald’s omega if item deleted** |
| --- | --- | --- | --- | --- |
| OSES_PS | OSES1 | 0.54 | 0.77 | 0.78 |
| OSES_PS | OSES2 | 0.43 | 0.80 | 0.81 |
| OSES_PS | OSES3 | 0.55 | 0.77 | 0.79 |
| OSES_PS | OSES4 | 0.74 | 0.70 | 0.72 |
| OSES_PS | OSES5 | 0.66 | 0.73 | 0.74 |
| OSES_CD | OSES6 | 0.60 | 0.82 | 0.83 |
| OSES_CD | OSES7 | 0.80 | 0.76 | 0.78 |
| OSES_CD | OSES8R | 0.24 | 0.89 | 0.89 |
| OSES_CD | OSES9 | 0.80 | 0.76 | 0.77 |
| OSES_CD | OSES10 | 0.78 | 0.77 | 0.79 |
| COPE_AV | COPE1 | 0.59 | 0.87 | 0.89 |
| COPE_AV | COPE2 | 0.75 | 0.84 | 0.86 |
| COPE_AV | COPE3 | 0.59 | 0.86 | 0.88 |
| COPE_AV | COPE4 | 0.90 | 0.80 | 0.82 |
| COPE_AV | COPE5 | 0.72 | 0.84 | 0.86 |
| COPE_PA | COPE6 | 0.29 | 0.63 | 0.68 |
| COPE_PA | COPE7 | 0.53 | 0.52 | 0.56 |
| COPE_PA | COPE8 | 0.57 | 0.50 | 0.58 |
| COPE_PA | COPE9 | 0.37 | 0.59 | 0.59 |
| COPE_PA | COPE10 | 0.30 | 0.62 | 0.63 |
| COPE_PA | COPE11 | 0.17 | 0.65 | 0.66 |
| COPE_PO | COPE12 | 0.63 | 0.74 | 0.77 |
| COPE_PO | COPE13 | 0.26 | 0.84 | 0.86 |
| COPE_PO | COPE14 | 0.76 | 0.69 | 0.72 |
| COPE_PO | COPE15 | 0.57 | 0.76 | 0.80 |
| COPE_PO | COPE16 | 0.72 | 0.71 | 0.73 |
| COPE_SSS | COPE17 | 0.54 | 0.74 | 0.75 |
| COPE_SSS | COPE18 | 0.58 | 0.72 | 0.75 |
| COPE_SSS | COPE19 | 0.51 | 0.75 | 0.76 |
| COPE_SSS | COPE20 | 0.55 | 0.74 | 0.78 |
| COPE_SSS | COPE21 | 0.62 | 0.71 | 0.76 |
| ACS_PTC | ACS1 | 0.66 | 0.76 | 0.77 |
| ACS_PTC | ACS2 | 0.68 | 0.75 | 0.78 |
| ACS_PTC | ACS3 | 0.73 | 0.73 | 0.76 |
| ACS_PTC | ACS4 | 0.50 | 0.83 | 0.84 |
| ACS_STC | ACS5 | 0.51 | 0.46 | 0.58 |
| ACS_STC | ACS6 | 0.27 | 0.63 | 0.70 |
| ACS_STC | ACS7 | 0.62 | 0.37 | 0.53 |
| ACS_STC | ACS8 | 0.24 | 0.68 | 0.69 |
| ACS_PRTC | ACS9 | 0.57 | 0.47 | 0.47 |
| ACS_PRTC | ACS10 | 0.46 | 0.61 | 0.62 |
| ACS_PRTC | ACS11 | 0.44 | 0.65 | 0.65 |
| MPFI_CF | MPFI1 | 0.44 | 0.74 | 0.80 |
| MPFI_CF | MPFI2 | 0.61 | 0.71 | 0.76 |
| MPFI_CF | MPFI3 | 0.43 | 0.74 | 0.78 |
| MPFI_CF | MPFI4 | 0.28 | 0.76 | 0.79 |
| MPFI_CF | MPFI5 | 0.41 | 0.74 | 0.79 |
| MPFI_CF | MPFI6 | 0.53 | 0.73 | 0.75 |
| MPFI_CF | MPFI7 | 0.37 | 0.75 | 0.76 |
| MPFI_CF | MPFI8 | 0.30 | 0.77 | 0.77 |
| MPFI_CF | MPFI9 | 0.35 | 0.75 | 0.76 |
| MPFI_CF | MPFI10 | 0.60 | 0.72 | 0.75 |
| MPFI_CINF | MPFI11 | 0.68 | 0.91 | 0.92 |
| MPFI_CINF | MPFI12 | 0.70 | 0.91 | 0.92 |
| MPFI_CINF | MPFI13 | 0.62 | 0.92 | 0.92 |
| MPFI_CINF | MPFI14 | 0.63 | 0.92 | 0.92 |
| MPFI_CINF | MPFI15 | 0.75 | 0.91 | 0.91 |
| MPFI_CINF | MPFI16 | 0.77 | 0.91 | 0.91 |
| MPFI_CINF | MPFI17 | 0.81 | 0.91 | 0.91 |
| MPFI_CINF | MPFI18 | 0.71 | 0.91 | 0.91 |
| MPFI_CINF | MPFI19 | 0.64 | 0.92 | 0.92 |
| MPFI_CINF | MPFI20 | 0.71 | 0.91 | 0.91 |
| SS_Friends | SS1 | 0.65 | 0.59 | ***** |
| SS_Friends | SS2 | 0.65 | 0.72 | ***** |
| SS_Family | SS3 | 0.71 | 0.67 | ***** |
| SS_Family | SS4 | 0.71 | 0.75 | ***** |
| SS_SM | SS5 | 0.79 | 0.87 | 0.87 |
| SS_SM | SS6 | 0.84 | 0.81 | 0.82 |
| SS_SM | SS7 | 0.78 | 0.88 | 0.88 |
| SS_Coll | SS8 | 0.74 | 0.80 | 0.80 |
| SS_Coll | SS9 | 0.76 | 0.78 | 0.78 |
| SS_Coll | SS10 | 0.71 | 0.83 | 0.83 |
| SS_ORG | SS11 | 0.88 | 0.87 | 0.89 |
| SS_ORG | SS12 | 0.86 | 0.91 | 0.91 |
| SS_ORG | SS13 | 0.86 | 0.90 | 0.91 |
| SEOR_SR | SEOR1 | 0.79 | 0.92 | 0.93 |
| SEOR_SR | SEOR2 | 0.81 | 0.92 | 0.92 |
| SEOR_SR | SEOR3 | 0.86 | 0.91 | 0.92 |
| SEOR_SR | SEOR4 | 0.76 | 0.93 | 0.93 |
| SEOR_SR | SEOR5 | 0.78 | 0.92 | 0.93 |
| SEOR_SR | SEOR6 | 0.85 | 0.92 | 0.92 |
| SEOR_IR | SEOR7 | 0.68 | 0.69 | 0.69 |
| SEOR_IR | SEOR8 | 0.66 | 0.71 | 0.71 |
| SEOR_IR | SEOR9 | 0.59 | 0.78 | 0.78 |

*Note.* *McDonald’s omega if item deleted was not computed for two-item subscales because deleting one item leaves a single-item scale.
