# Supplementary material for: Change Adaptation Resilience Evaluation Questionnaire for Industry 5.0 Among Workers in High-Tech Manufacturing Companies: Questionnaire Development and Pilot Evaluation Study
Source: JMIR Hum Factors. 2026 Aug 28;13:e83754. doi: 10.2196/83754 (PMC13524363; doi:10.2196/83754)
Supplement: Supplementary file 2 [file humanfactors-v13-e83754-s002.docx]

**Table S1.** CARE-QI 5.0 structure and items selected (N = 84 items)

| **Two-high order dimensions** | **Factors** | **Sub-factors** | **Item Italian Version CARE QI 5.0** | | **Item English version CARE QI 5.0** | **Adaptation** |
| --- | --- | --- | --- | --- | --- | --- |
| **Individual Resilience** | **Self-efficacy (OSES)** | **Problem solving** | 1 | Se si verificano dei problemi sul lavoro, di solito riesco a pensare a qualcosa per risolverli | If problems arise at work, I usually manage to come up with something to solve them | Minor linguistic and semantic refinement; no substantive change to the original construct |
|  |  |  | 2 | Quando affronto le difficoltà nel mio lavoro riesco a rimanere tranquillo/a perché posso contare sulle mie abilità | When I face difficulties at work, I remain calm because I can rely on my abilities |  |
|  |  |  | 3 | Quando ho di fronte un problema sul lavoro, di solito riesco a trovare diverse soluzioni | When I have a problem at work, I usually find several solutions |  |
|  |  |  | 4 | Sono sicuro/a di poter trattare efficacemente gli imprevisti del mio lavoro | I am confident that I can effectively handle unforeseen events at work |  |
|  |  |  | 5 | Le mie esperienze lavorative passate mi sono state utili a prepararmi per eventuali problemi che posso incontrare nel mio percorso lavorativo | My past work experiences have helped prepare me for any problems I may encounter |  |
|  |  | **Confidence to persevere in the face of difficulties** | 6 | Quando capitano problemi imprevisti sul lavoro, non li gestisco molto bene | When unexpected problems arise at work, I don't handle them very well | Minor linguistic and semantic refinement; no substantive change to the original construct |
|  |  |  | 7 | Evito di provare ad imparare cose nuove nel mio lavoro, quando queste appaiono troppo difficili per me | I avoid trying to learn new things at work when they seem too difficult for me |  |
|  |  |  | 8 | Quando a lavoro qualcosa non funziona subito, cerco di impegnarmi di più | When something doesn't work immediately at work, I try harder |  |
|  |  |  | 9 | Quando qualcosa non funziona bene nel mio lavoro, rinuncio facilmente | When something doesn’t work well at work, I give up easily |  |
|  |  |  | 10 | Non mi sembra di essere capace di occuparmi della maggior parte dei problemi che sorgono nel mio lavoro | I don’t feel capable of handling most of the problems that arise at work |  |
|  | **Coping Orientation to Problems Experienced (COPE)** | **Avoidance strategies** | 11 | Quando ho un problema al lavoro, riconosco che non posso farci niente e abbandono ogni tentativo di agire | When I have a problem at work, I recognize that there's nothing I can do and I give up trying | Contextual adaptation to work environment; linguistic simplification; preservation of original coping strategy content |
|  |  |  | 12 | Quando ho un problema al lavoro, tendo a fantasticare per distrarmi | When I have a problem at work, I tend to fantasize to distract myself |  |
|  |  |  | 13 | Quando ho un problema al lavoro, mi rifiuto di credere che ciò sia accaduto | When I have a problem at work, I refuse to believe it happened |  |
|  |  |  | 14 | Quando ho un problema al lavoro, mi comporto come se non fosse mai accaduto | When I have a problem at work, I act as if it never happened |  |
|  |  |  | 15 | Quando ho un problema al lavoro, non mi impegno più di tanto per risolverlo | When I have a problem at work, I don’t try very hard to solve it |  |
|  |  | **Positive attitude** | 16 | Quando ho un problema al lavoro, imparo a conviverci | When I have a problem at work, I learn to live with it | Contextual adaptation to work environment; linguistic simplification; minor semantic refinement |
|  |  |  | 17 | Quando ho un problema al lavoro, accetto la realtà dei fatti | When I have a problem at work, I accept the reality of the situation |  |
|  |  |  | 18 | Quando ho un problema al lavoro, cerco di abituarmi all'idea che ciò è successo | When I have a problem at work, I try to get used to the idea that it happened |  |
|  |  |  | 19 | Quando ho un problema al lavoro, cerco qualcosa di positivo in ciò che è accaduto | When I have a problem at work, I look for something positive in what happened |  |
|  |  |  | 20 | Quando ho un problema al lavoro, cerco di utilizzare questa esperienza per crescere come persona | When I have a problem at work, I try to use the experience to grow as a person |  |
|  |  |  | 21 | Quando ho un problema al lavoro, tento di imparare qualcosa di buono dall'esperienza | When I have a problem at work, I try to learn something good from the experience |  |
|  |  | **Problem-oriented** | 22 | Quando ho un problema al lavoro, mi concentro nel gestire questo problema e, se necessario, metto da parte le altre cose che ho da fare | When I have a problem at work, I focus on dealing with it and, if necessary, set aside other tasks | Contextual adaptation to work environment; linguistic simplification; increased action-oriented clarity |
|  |  |  | 23 | Quando ho un problema al lavoro, cerco in tutti i modi di impedire che altre cose interferiscano con i miei sforzi di affrontarlo | When I have a problem at work, I do my best to prevent other things from interfering with dealing with it |  |
|  |  |  | 24 | Quando ho un problema al lavoro, mi impegno al massimo per agire sulla situazione | When I have a problem at work, I give my best effort to act on the situation |  |
|  |  |  | 25 | Quando ho un problema al lavoro rifletto intensamente su quali mosse fare | When I have a problem at work, I think deeply about what steps to take |  |
|  |  |  | 26 | Quando ho un problema al lavoro faccio quello che deve essere fatto, un passo alla volta | When I have a problem at work, I do what must be done, one step at a time |  |
|  |  | **Seeking Social Support** | 27 | Quando ho un problema al lavoro, cerco di farmi consigliare da qualcuno sul da farsi | When I have a problem at work, I seek advice from someone on what to do | Contextual adaptation to work environment; linguistic simplification; preservation of interpersonal coping processes |
|  |  |  | 28 | Quando ho un problema al lavoro parlo con qualcuno per fare qualcosa di concreto per risolvere il problema | When I have a problem at work, I talk to someone to do something concrete to solve it |  |
|  |  |  | 29 | Quando ho un problema al lavoro chiedo alle persone come hanno agito di fronte ad esperienze simili | When I have a problem at work, I ask people how they dealt with similar experiences |  |
|  |  |  | 30 | Quando ho un problema al lavoro, cerco la comprensione e la solidarietà di qualcuno | When I have a problem at work, I seek understanding and solidarity from someone |  |
|  |  |  | 31 | Quando ho un problema al lavoro, cerco sostegno morale dai miei amici e dai parenti | When I have a problem at work, I seek moral support from my friends and relatives |  |
|  | **Change management and adaptation (ACS)** | **Predisposition to change** | 32 | Nelle mie attività lavorative, individuo facilmente soluzioni alternative ai problemi che incontro | In my work activities, I easily find alternative solutions to the problems I encounter | Contextual adaptation to work environment; linguistic simplification; semantic refinement for workplace applicability |
|  |  |  | 33 | Pensare a nuovi modi per affrontare il lavoro che devo fare è facile per me | Thinking of new modalities to tackle my work is easy for me |  |
|  |  |  | 34 | Di fronte ad un cambiamento nel mio luogo di lavoro so vedere le cose da molteplici prospettive | Faced with change at work, I can see things from multiple perspectives |  |
|  |  |  | 35 | Sono in grado di cogliere le opportunità che mi si presentano sul lavoro | I am able to seize opportunities that arise at work |  |
|  |  | **Seeking to change** | 36 | Nella mia vita lavorativa, sono sempre in cerca di cambiamenti. | In my working life, I am always looking for changes | Contextual adaptation to work environment; linguistic simplification; minor semantic rephrasing |
|  |  |  | 37 | Normalmente cerco modi diversi per fare le stesse cose della mia vita lavorativa | I usually look for different ways to do the same things in my work life |  |
|  |  |  | 38 | Nella mia vita lavorativa, sono in cerca di cambiamenti anche quando le cose vanno bene | In my working life, I seek changes even when things are going well |  |
|  |  |  | 39 | Anche se vedo benefici di questo lavoro, vorrei cambiarlo | Even if I see the benefits of this job, I would like to change it |  |
|  |  | **Positive reaction to change** | 40 | A lavoro riesco a trovare aspetti positivi nei cambiamenti che potrebbero sembrare negativi | At work, I manage to find positive aspects in changes that might seem negative | Contextual adaptation to work environment; linguistic simplification; semantic refinement |
|  |  |  | 41 | Sono in grado di tollerare gli aspetti negativi dei cambiamenti che si verificano sul lavoro | I can tolerate the negative aspects of changes that occur at work |  |
|  |  |  | 42 | Riesco a trovare aspetti diversi alle mansioni che faccio da tanto tempo | I manage to find different aspects in tasks I’ve been doing for a long time |  |
|  | **Cognitive Flexibility (MPFI)** | **Cognitive flexibility** | 43 | Sono disposto/a ad osservare pensieri e sentimenti spiacevoli senza interferire con essi. | I am willing to observe unpleasant thoughts and feelings without interfering with them. | Tense shift from past/retrospective to present/dispositional framing; minor linguistic and semantic refinement; preservation of original construct content |
|  |  |  | 44 | Cerco di far pace con pensieri e sentimenti negativi anziché respingerli | I try to make peace with negative thoughts and feelings rather than push them away. |  |
|  |  |  | 45 | Faccio spazio ad emozioni e a pensieri negativi, vivendoli appieno anziché respingerli | I make room for negative emotions and thoughts, fully experiencing them instead of rejecting them. |  |
|  |  |  | 46 | Quando ho un'emozione o un pensiero negativo, cerco di dargli importanza anziché ignorarlo | When I have a negative emotion or thought, I try to give it importance rather than ignore it. |  |
|  |  |  | 47 | Mi apro a tutti i sentimenti, sia buoni che cattivi | I open myself up to all feelings, both good and bad. |  |
|  |  |  | 48 | Anche quando i miei sforzi non sono riconosciuti, ho comunque continuato ad agire verso ciò che per me è importante | Even when my efforts are not recognized, I still continue to act toward what is important to me. |  |
|  |  |  | 49 | Anche nei momenti più difficili, riesco a procedere verso quello che ritengo più importante nella vita | Even in the most difficult moments, I manage to move toward what I consider most important in life. |  |
|  |  |  | 50 | Anche quando la vita diventa stressante e frenetica, continuo ad impegnarmi per le cose che sono importanti per me | Even when life becomes stressful and hectic, I keep striving for the things that matter to me. |  |
|  |  |  | 51 | Non lascio che gli ostacoli mi frenino nel progredire verso ciò che veramente voglio nella vita | I don't let obstacles stop me from progressing toward what I truly want in life. |  |
|  |  |  | 52 | Non lascio che paure o dubbi si intromettano tra me e i miei obiettivi | I don't let fears or doubts get between me and my goals. |  |
|  |  | **Cognitive Inflexibility** | 53 | I pensieri e i sentimenti negativi tendono a restarmi appiccicati addosso a lungo | Negative thoughts and feelings tend to stick with me for a long time. | Tense shift from past/retrospective to present/dispositional framing; minor linguistic and semantic refinement; preservation of original construct content |
|  |  |  | 54 | I pensieri angoscianti tendono a girarmi in testa come un disco rotto | Distressing thoughts tend to spin in my head like a broken record. |  |
|  |  |  | 55 | È molto facile restare intrappolati in pensieri e sentimenti negativi quando si verificano problemi inaspettati | It’s very easy to get trapped in negative thoughts and feelings when unexpected problems occur. |  |
|  |  |  | 56 | Quando ho pensieri o sentimenti negativi, è molto difficile riuscire a guardare oltre | When I have negative thoughts or feelings, it's very difficult to see beyond them. |  |
|  |  |  | 57 | Quando succede qualcosa di brutto, è difficile smettere di pensarci | When something bad happens, it’s hard to stop thinking about it. |  |
|  |  |  | 58 | I sentimenti negativi spesso mi bloccano, non lasciandomi agire | Negative feelings often paralyze me and prevent me from acting. |  |
|  |  |  | 59 | I sentimenti negativi ostacolano facilmente i miei piani | Negative feelings easily get in the way of my plans. |  |
|  |  |  | 60 | Quando sono turbato/a mi blocco e non riesco ad agire | When I’m upset, I freeze and can’t take action. |  |
|  |  |  | 61 | Le esperienze negative mi distolgono da ciò che per me è veramente importante | Negative experiences distract me from what really matters to me. |  |
|  |  |  | 62 | I pensieri e i sentimenti negativi mi creano difficoltà nel dare significato alla mia vita | Negative thoughts and feelings make it hard for me to give meaning to my life. |  |
| **Organizational Resilience (Contextual)** | **Social Support** | **Friends** | 63 | I miei amici cercano di aiutarmi quando parlo delle mie cose di lavoro | My friends try to help me when I talk about work-related matters | Contextual adaptation to work-related domain; minor linguistic simplification |
|  |  |  | 64 | Quando qualcosa va storto sul lavoro posso contare sui miei amici | When something goes wrong at work, I can count on my friends |  |
|  |  | **Family** | 65 | La mia famiglia mi sostiene emotivamente e mi aiuta per le cose di lavoro | My family supports me emotionally and helps with work-related matters | Contextual adaptation to work-related domain; minor linguistic simplification |
|  |  |  | 66 | Posso parlare dei miei problemi lavorativi in famiglia | I can talk about my work problems with my family |  |
|  |  | **Supervisor** | 67 | Posso fare affidamento sul/sulla mio/a diretto/a responsabile nel caso avessi problemi di lavoro | I can rely on my direct supervisor if I have problems at work | Linguistic simplification; minor semantic refinement; preservation of original construct content |
|  |  |  | 68 | Il/La mio/a diretto/a responsabile mi incoraggia nel lavoro | My direct supervisor encourages me in my work |  |
|  |  |  | 69 | Il/La mio/a diretto/a responsabile mi fornisce ciò di cui ho bisogno per svolgere efficacemente il mio lavoro | My direct supervisor provides what I need to perform my job effectively |  |
|  |  | **Colleagues** | 70 | Se il lavoro diventa difficile, posso contare sull’aiuto dei/delle miei/mie colleghi/colleghe | If work becomes difficult, I can count on the help of my colleagues | Linguistic simplification; minor semantic refinement; preservation of original construct content |
|  |  |  | 71 | I/Le colleghi/colleghe mi danno l’aiuto e il supporto di cui ho bisogno | My colleagues give me the help and support I need |  |
|  |  |  | 72 | I/Le colleghi/colleghe sono disponibili ad ascoltare i miei problemi di lavoro | My colleagues are willing to listen to my work problems |  |
|  |  | **Organization** | 73 | La mia azienda tiene in gran considerazione i miei obiettivi e i miei valori | My company values my goals and values highly | Linguistic simplification; preservation of original organizational support construct |
|  |  |  | 74 | Se ho un problema, la mia azienda è pronta ad aiutarmi | If I have a problem, my company is ready to help me |  |
|  |  |  | 75 | La mia azienda si preoccupa davvero del mio benessere | My company genuinely cares about my well-being |  |
|  | **Organizational Resilience (SEOR)** | **Skill-based resilience** | 76 | La mia azienda usa le proprie risorse per addestrare e formare il personale allo svolgimento del lavoro | My company uses its resources to train and educate staff for the job | Contextual lexical refinement (organization to company); linguistic simplification; minor semantic adaptation for manufacturing relevance |
|  |  |  | 77 | Il personale ha una formazione e un’esperienza più che sufficienti per il lavoro che fa | Staff have more than sufficient training and experience for their work |  |
|  |  |  | 78 | La mia azienda è attivamente interessata a sviluppare le competenze e le conoscenze delle persone che ci lavorano | My company is actively interested in developing the skills and knowledge of its people |  |
|  |  |  | 79 | La mia azienda promuove l’assegnazione di incarichi che permettono di misurarsi con delle sfide | My company promotes task assignments that allow people to face challenges |  |
|  |  |  | 80 | Le persone che lavorano nella mia azienda riescono ad applicare le proprie conoscenze al lavoro in modo creativo | People in my company apply their knowledge to their work creatively |  |
|  |  |  | 81 | La mia azienda si impegna a promuovere l'uso di strategie innovative per risolvere i problemi | My company is committed to promoting innovative strategies to solve problems |  |
|  |  | **Individual-based resilience** | 82 | Nella mia azienda le persone imparano dai propri errori | In my company, people learn from their mistakes | Minimal lexical adaptation (organization to company); preservation of original construct content |
|  |  |  | 83 | Nella mia azienda le persone fanno affidamento le une sulle altre | In my company, people rely on each other |  |
|  |  |  | 84 | Nella mia azienda la maggior parte delle persone ha le competenze necessarie ad affrontare i problemi inattesi che si verificano | In my company, most people have the skills needed to deal with unexpected problems |  |
