# Supplementary material for: Change Adaptation Resilience Evaluation Questionnaire for Industry 5.0 Among Workers in High-Tech Manufacturing Companies: Questionnaire Development and Pilot Evaluation Study
Source: JMIR Hum Factors. 2026 Aug 28;13:e83754. doi: 10.2196/83754 (PMC13524363; doi:10.2196/83754)
Supplement: Supplementary file 3 [file humanfactors-v13-e83754-s003.docx]

**Table S2.** Items’ Descriptive Statistics

|  |  | **Original Item** | **CARE-QI 5.0 Item** | **min** | **max** | **mean** | **SD** | **skewness** | **kurtosis** |
| --- | --- | --- | --- | --- | --- | --- | --- | --- | --- |
| **Self-efficacy (OSES)** | **Problem solving (OSES-PS)** | OSES1 | 1 | 1 | 5 | 4.05 | 0.91 | -1.24 | 2.09 |
|  |  | OSES2 | 2 | 1 | 5 | 3.98 | 0.73 | -1.46 | 5.47 |
|  |  | OSES3 | 3 | 1 | 5 | 3.89 | 0.62 | -0.55 | 1.41 |
|  |  | OSES4 | 4 | 1 | 5 | 3.82 | 0.90 | -1.25 | 2.84 |
|  |  | OSES5 | 5 | 1 | 5 | 3.89 | 0.95 | -0.80 | 0.77 |
|  |  | **OSES-PS** |  | 1 | 5 | 3.92 | 0.62 | -0.47 | 0.90 |
|  | **Confidence to persevere in the face of difficulties (OSES-CD)** | OSES6 | 6 | 1 | 5 | 2.23 | 1.05 | 0.90 | 0.60 |
|  |  | OSES7 | 7 | 1 | 5 | 1.98 | 0.98 | 0.99 | 0.91 |
|  |  | OSES8R | 8R | 1 | 5 | 2.00 | 0.65 | -0.54 | 1.36 |
|  |  | OSES9 | 9 | 1 | 5 | 2.05 | 1.18 | 1.24 | 0.86 |
|  |  | OSES10 | 10 | 1 | 5 | 2.09 | 1.05 | 1.07 | 1.03 |
|  |  | **OSES-CD** |  | 1 | 5 | 2.07 | 0.78 | 0.70 | 0.18 |
| **Coping Orientation to Problems Experienced (COPE)** | **Avoidance strategies** | COPE1 | 11 | 1 | 5 | 2.27 | 1.11 | 0.72 | -0.09 |
|  |  | COPE2 | 12 | 1 | 5 | 1.80 | 0.90 | 1.42 | 2.67 |
|  |  | COPE3 | 13 | 1 | 5 | 2.00 | 1.14 | 1.18 | 0.69 |
|  |  | COPE4 | 14 | 1 | 5 | 1.84 | 0.94 | 1.39 | 2.27 |
|  |  | COPE5 | 15 | 1 | 5 | 1.80 | 1.03 | 1.65 | 2.87 |
|  |  | **COPE-AV** |  | 1 | 5 | 1.94 | 0.84 | 1.47 | 3.31 |
|  | **Positive attitude** | COPE6 | 16 | 1 | 5 | 2.48 | 1.19 | 0.45 | -0.53 |
|  |  | COPE7 | 17 | 1 | 5 | 2.89 | 1.13 | 0.03 | -0.76 |
|  |  | COPE8 | 18 | 1 | 5 | 3.02 | 1.19 | 0.41 | -0.98 |
|  |  | COPE9 | 19 | 1 | 5 | 3.61 | 0.99 | -0.48 | -0.14 |
|  |  | COPE10 | 20 | 1 | 5 | 4.23 | 0.57 | 0.02 | -0.20 |
|  |  | COPE11 | 21 | 1 | 5 | 4.20 | 0.67 | -0.75 | 1.57 |
|  |  | **COPE-PA** |  | 1 | 5 | 3.41 | 0.59 | 0.71 | 0.84 |
|  | **Problem-oriented** | COPE12 | 22 | 1 | 5 | 3.68 | 0.93 | -0.56 | 0.39 |
|  |  | COPE13 | 23 | 1 | 5 | 3.64 | 0.72 | 0.28 | -0.42 |
|  |  | COPE14 | 24 | 1 | 5 | 4.11 | 0.81 | -1.30 | 3.62 |
|  |  | COPE15 | 25 | 1 | 5 | 4.11 | 0.69 | -0.60 | 0.93 |
|  |  | COPE16 | 26 | 1 | 5 | 4.14 | 0.73 | -1.70 | 6.76 |
|  |  | **COPE-PO** |  | 1 | 5 | 3.94 | 0.58 | -0.01 | 0-13 |
|  | **Seeking Social Support** | COPE17 | 27 | 1 | 5 | 3.61 | 0.81 | 0.02 | -0.45 |
|  |  | COPE18 | 28 | 1 | 5 | 3.80 | 0.77 | -0.28 | -0.07 |
|  |  | COPE19 | 29 | 1 | 5 | 3.95 | 0.68 | -0.41 | 0.62 |
|  |  | COPE20 | 30 | 1 | 5 | 3.32 | 1.01 | -0.41 | -0.23 |
|  |  | COPE21 | 31 | 1 | 5 | 3.32 | 0.96 | -0.36 | 0.20 |
|  |  | **COPE-SSS** |  | 1 | 5 | 3.60 | 0.62 | 0.33 | 0.43 |
| **Change management and adaptation (ACS)** | **Predisposition to change** | ACS1 | 32 | 1 | 5 | 3.75 | 0.94 | -0.69 | 0.53 |
|  |  | ACS2 | 33 | 1 | 5 | 3.82 | 0.84 | -0.37 | -0.29 |
|  |  | ACS3 | 34 | 1 | 5 | 3.75 | 0.87 | -0.83 | 1.37 |
|  |  | ACS4 | 35 | 1 | 5 | 3.86 | 0.77 | -0.41 | -0.13 |
|  |  | **ACS-PTC** |  | 1 | 5 | 3.80 | 0.69 | -0.28 | -0.06 |
|  | **Seeking to change** | ACS5 | 36 | 1 | 5 | 3.34 | 0.94 | -0.22 | -0.25 |
|  |  | ACS6 | 37 | 1 | 5 | 3.64 | 0.87 | -0.33 | -0.74 |
|  |  | ACS7 | 38 | 1 | 5 | 2.98 | 0.98 | 0.20 | -0.23 |
|  |  | ACS8 | 39 | 1 | 5 | 2.61 | 1.06 | 0.36 | -0.40 |
|  |  | **ACS-STC** |  | 1 | 5 | 3.14 | 0.66 | 0.56 | 0.70 |
|  | **Positive reaction to change** | ACS9 | 40 | 1 | 5 | 3.55 | 0.85 | 0.09 | -0.53 |
|  |  | ACS10 | 41 | 1 | 5 | 3.50 | 0.85 | 0.000 | -0.51 |
|  |  | ACS11 | 42 | 1 | 5 | 3.55 | 0.98 | -0.69 | 0.62 |
|  |  | ACS12* |  | 1 | 5 | 3.77 | 0.83 | -1.32 | 2.50 |
|  |  | **ACS-PRTC** |  | 1 | 5 | 3.59 | 0.62 | 0.03 | 1.66 |
| **Cognitive Flexibility (MPFI)** | **Cognitive Flexibility** | MPFI1 | 43 | 1 | 5 | 3.39 | 0.84 | -0.36 | 0.54 |
|  |  | MPFI2 | 44 | 1 | 5 | 3.52 | 0.82 | -0.74 | 1.09 |
|  |  | MPFI3 | 45 | 1 | 5 | 2.98 | 0.98 | -0.42 | -0.29 |
|  |  | MPFI4 | 46 | 1 | 5 | 3.20 | 0.82 | -0.40 | -0.01 |
|  |  | MPFI5 | 47 | 1 | 5 | 3.50 | 0.85 | -1.19 | 1.96 |
|  |  | MPFI6 | 48 | 1 | 5 | 4.14 | 0.55 | 0.07 | 0.26 |
|  |  | MPFI7 | 49 | 1 | 5 | 4.18 | 0.50 | 0.40 | 0.52 |
|  |  | MPFI8 | 50 | 1 | 5 | 4.25 | 0.58 | -0.05 | -0.33 |
|  |  | MPFI9 | 51 | 1 | 5 | 4.18 | 0.62 | -0.13 | -0.40 |
|  |  | MPFI10 | 52 | 1 | 5 | 3.75 | 0.75 | -0.24 | -0.06 |
|  |  | **MPFI-CF** |  | 1 | 5 | 3.71 | 0.42 | 0.40 | 1.39 |
|  | **Cognitive Inflexibility** | MPFI11 | 53 | 1 | 5 | 2.93 | 1.09 | 0.37 | -0.33 |
|  |  | MPFI12 | 54 | 1 | 5 | 2.98 | 1.11 | 0.37 | -0.78 |
|  |  | MPFI13 | 55 | 1 | 5 | 3.25 | 0.97 | -0.05 | -0.49 |
|  |  | MPFI14 | 56 | 1 | 5 | 2.93 | 1.04 | 0.27 | -0.76 |
|  |  | MPFI15 | 57 | 1 | 5 | 3.30 | 1.05 | 0.13 | -0.67 |
|  |  | MPFI16 | 58 | 1 | 5 | 2.59 | 1.041 | 0.65 | -0.32 |
|  |  | MPFI17 | 59 | 1 | 5 | 2.61 | 0.99 | 0.57 | -0.01 |
|  |  | MPFI18 | 60 | 1 | 5 | 2.57 | 1.04 | 0.45 | -0.20 |
|  |  | MPFI19 | 61 | 1 | 5 | 2.43 | 0.97 | 1.07 | 0.81 |
|  |  | MPFI20 | 62 | 1 | 5 | 2.52 | 1.15 | 0.66 | -0.39 |
|  |  | **MPFI-CINF** |  | 1 | 5 | 2.81 | 0.80 | 0.48 | 0.50 |
| **Social Support (SS)** | **Friends** | SS1 | 63 | 1 | 5 | 3.61 | 0.95 | -0.17 | -0.80 |
|  |  | SS2 | 64 | 1 | 5 | 3.45 | 1.04 | -0.39 | -0.15 |
|  |  | **SS-Friends** |  | 1 | 5 | 3.53 | 0.90 | -0.08 | -0.62 |
|  | **Family** | SS3 | 65 | 1 | 5 | 3.75 | 0.89 | -0.71 | 0.97 |
|  |  | SS4 | 66 | 1 | 5 | 3.89 | 0.95 | -1.50 | 2.82 |
|  |  | **SS-Family** |  | 1 | 5 | 3.82 | 0.85 | -1.06 | 2.11 |
|  | **Supervisor/Manager** | SS5 | 67 | 1 | 5 | 3.50 | 1.13 | -0.61 | -0.20 |
|  |  | SS6 | 68 | 1 | 5 | 3.36 | 1.08 | -0.67 | -0.27 |
|  |  | SS7 | 69 | 1 | 5 | 3.48 | 0.93 | -1.12 | 1.71 |
|  |  | **SS-S/M** |  | 1 | 5 | 3.45 | 0.96 | -0.68 | 0.19 |
|  | **Colleagues** | SS8 | 70 | 1 | 5 | 3.82 | 0.84 | -0.61 | 1.39 |
|  |  | SS9 | 71 | 1 | 5 | 3.57 | 0.87 | -0.33 | 0.61 |
|  |  | SS10 | 72 | 1 | 5 | 3.59 | 0.97 | -0.83 | 0.82 |
|  |  | **SS-Coll** |  | 1 | 5 | 3.66 | 0.79 | -0.31 | 0.74 |
|  | **Organization** | SS11 | 73 | 1 | 5 | 2.64 | 1.18 | 0.06 | -0.94 |
|  |  | SS12 | 74 | 1 | 5 | 3.00 | 0.94 | 0.000 | -0.28 |
|  |  | SS13 | 75 | 1 | 5 | 3.00 | 1.08 | -0.35 | -0.57 |
|  |  | **SS-Org** |  | 1 | 5 | 2.88 | 1.00 | -0.02 | -0.53 |
| **Organizational Resilience (SEOR)** | **Skill-based Resilience** | SEOR1 | 76 | 1 | 5 | 3.30 | 1.17 | -0.61 | -0.40 |
|  |  | SEOR2 | 77 | 1 | 5 | 3.20 | 0.98 | -0.59 | -0.02 |
|  |  | SEOR3 | 78 | 1 | 5 | 2.95 | 1.18 | -0.18 | -0.95 |
|  |  | SEOR4 | 79 | 1 | 5 | 2.80 | 0.95 | -0.24 | -0.12 |
|  |  | SEOR5 | 80 | 1 | 5 | 2.95 | 1.01 | -0.05 | -0.58 |
|  |  | SEOR6 | 81 | 1 | 5 | 2.93 | 1.07 | -0.34 | -0.83 |
|  |  | **SEOR-SR** |  | 1 | 5 | 3.04 | 0.92 | -0.19 | -0.65 |
|  | **Individual-based resilience** | SEOR7 | 82 | 1 | 5 | 3.07 | 1.04 | -0.40 | -0.45 |
|  |  | SEOR8 | 83 | 1 | 5 | 2.95 | 1.12 | -0.22 | -0.92 |
|  |  | SEOR9 | 84 | 1 | 5 | 3.09 | 1.12 | -0.19 | -0.62 |
|  |  | **SEOR-IR** |  | 1 | 5 | 3.02 | 0.92 | -0.44 | -0.18 |

*Note.* *Not included in the final item pool; R = Reversed.
