# Supplementary material for: Change Adaptation Resilience Evaluation Questionnaire for Industry 5.0 Among Workers in High-Tech Manufacturing Companies: Questionnaire Development and Pilot Evaluation Study
Source: JMIR Hum Factors. 2026 Aug 28;13:e83754. doi: 10.2196/83754 (PMC13524363; doi:10.2196/83754)
Supplement: Supplementary file 4 [file humanfactors-v13-e83754-s004.docx]

**Table S3.** Inter-item correlations within subscales with their total scales. *p<0.05; **p<0.001

1. **INDIVIDUAL FACTORS**

- **Self-efficacy scale (OSES)**

| **Problem Solving (OSES_PS)** | | | | | | |
| --- | --- | --- | --- | --- | --- | --- |
|  | OSES1 | OSES2 | OSES3 | OSES4 | OSES5 | OSES_PS |
| OSES1 | 1 |  |  |  |  |  |
| OSES2 | .169 | 1 |  |  |  |  |
| OSES3 | .440** | .406** | 1 |  |  |  |
| OSES4 | .528** | .467** | .397** | 1 |  |  |
| OSES5 | .471** | .333* | .416** | .653** | 1 |  |
| OSES_PS | .732** | .609** | .674** | .849** | .805** | 1 |
| **Confidence to persevere in the face of difficulties (OSES_CD)** | | | | | | |
|  | OSES6 | OSES7 | OSES9 | OSES10 | OSES8R | OSES_CD |
| OSES6 | 1 |  |  |  |  |  |
| OSES7 | .615** | 1 |  |  |  |  |
| OSES9 | .514** | .801** | 1 |  |  |  |
| OSES10 | .580** | .721** | .697** | 1 |  |  |
| OSES8R | .102 | .147 | .332* | .237 | 1 |  |
| OSES_CD | .757** | .883** | .891** | .863** | .397** | 1 |

| - **Coping Orientation to Problems Experienced scale (COPE)** | | | | | | |  |
| --- | --- | --- | --- | --- | --- | --- | --- |
| **Avoidance strategies (COPE_AV)** | | | | | | |  |
|  | COPE1 | COPE2 | COPE3 | COPE4 | COPE5 | COPE_AV |  |
| COPE1 | 1 |  |  |  |  |  |  |
| COPE2 | .500** | 1 |  |  |  |  |  |
| COPE3 | .346* | .585** | 1 |  |  |  |  |
| COPE4 | .601** | .756** | .738** | 1 |  |  |  |
| COPE5 | .603** | .657** | .397** | .764** | 1 |  |  |
| COPE_AV | .752** | .839** | .754** | .936** | .827** | 1 |  |
| **Positive attitude (COPE_PA)** | | | | | | |  |
|  | COPE6 | COPE7 | COPE8 | COPE9 | COPE10 | COPE11 | COPE_PA |
| COPE6 | 1 |  |  |  |  |  |  |
| COPE7 | .338* | 1 |  |  |  |  |  |
| COPE8 | .366* | .582** | 1 |  |  |  |  |
| COPE9 | .286 | .154 | .364* | 1 |  |  |  |
| COPE10 | -.041 | .103 | .088 | .234 | 1 |  |  |
| COPE11 | -.277 | .268 | .018 | .081 | .800** | 1 |  |
| COPE_PA | .595** | .742** | .768** | .603** | .424** | .335* | 1 |
| **Problem-oriented (COPE_PO)** | | | | | | |  |
|  | COPE12 | COPE13 | COPE14 | COPE15 | COPE16 | COPE_PO |  |
| COPE12 | 1 |  |  |  |  |  |  |
| COPE13 | .164 | 1 |  |  |  |  |  |
| COPE14 | .626** | .227 | 1 |  |  |  |  |
| COPE15 | .381** | .361* | .516** | 1 |  |  |  |
| COPE16 | .671** | .091 | .792** | .475** | 1 |  |  |
| COPE_PO | .801** | .475** | .863** | .716** | .828** | 1 |  |
| **Seeking Social Support (COPE_SSS)** | | | | | | |  |
|  | COPE17 | COPE18 | COPE19 | COPE20 | COPE21 | COPE_SSS |  |
| COPE17 | 1 |  |  |  |  |  |  |
| COPE18 | .661** | 1 |  |  |  |  |  |
| COPE19 | .467** | .598** | 1 |  |  |  |  |
| COPE20 | .251 | .258 | .285 | 1 |  |  |  |
| COPE21 | .332* | .349* | .223 | .785** | 1 |  |  |
| COPE_SSS | .708** | .739** | .646** | .762** | .790** | 1 |  |

- **Change management and adaptation scale (ACS)**

| **Predisposition to change (ACS_PTC)** | | | | | | |
| --- | --- | --- | --- | --- | --- | --- |
|  | ACS1 | ACS2 | ACS3 | ACS4 | ACS_PTC |  |
| ACS1 | 1 |  |  |  |  |  |
| ACS2 | .566** | 1 |  |  |  |  |
| ACS3 | .740** | .575** | 1 |  |  |  |
| ACS4 | .400** | .619** | .417** | 1 |  |  |
| ACS_PTC | .845** | .842** | .845** | .728** | 1 |  |
| **Seeking to change (ACS_STC)** | | | | | |  |
|  | ACS5 | ACS6 | ACS7 | ACS8 | ACS_STC |  |
| ACS5 | 1 |  |  |  |  |  |
| ACS6 | .366* | 1 |  |  |  |  |
| ACS7 | .580** | .368* | 1 |  |  |  |
| ACS8 | .199 | .041 | .365* | 1 |  |  |
| ACS_STC | .757** | .612** | .827** | .601** | 1 |  |
| **Positive reaction to change (ACS_PRT)** | | | | | |  |
|  | ACS9 | ACS10 | ACS11 | ACS_PRT |  |  |
| ACS9 | 1 |  |  |  |  |  |
| ACS10 | .493** | 1 |  |  |  |  |
| ACS11 | .441** | .303* | 1 |  |  |  |
| ACS_PRT | .794** | .720** | .709** | 1 |  |  |

- **Cognitive Flexibility-Inflexibility scale (MPFI)**

| **Cognitive flexibility (MPFI_CF)** | | | | | | | | | | | |
| --- | --- | --- | --- | --- | --- | --- | --- | --- | --- | --- | --- |
|  | MPFI1 | MPFI2 | MPFI3 | MPFI4 | MPFI5 | MPFI6 | MPFI7 | MPFI8 | MPFI9 | MPFI10 | MPFI_CF |
| MPFI1 | 1 |  |  |  |  |  |  |  |  |  |  |
| MPFI2 | .617** | 1 |  |  |  |  |  |  |  |  |  |
| MPFI3 | .272 | .230 | 1 |  |  |  |  |  |  |  |  |
| MPFI4 | .041 | .235 | .619** | 1 |  |  |  |  |  |  |  |
| MPFI5 | .476** | .494** | .439** | .214 | 1 |  |  |  |  |  |  |
| MPFI6 | .280 | .305* | .293* | .142 | .169 | 1 |  |  |  |  |  |
| MPFI7 | -.017 | .150 | -.031 | -.031 | -.100 | .657** | 1 |  |  |  |  |
| MPFI8 | -.026 | .147 | -.103 | -.102 | -.012 | .264 | .655** | 1 |  |  |  |
| MPFI9 | -.013 | .256 | -.063 | -.025 | -.124 | .396** | .724** | .782** | 1 |  |  |
| MPFI10 | .253 | .355* | .367* | .190 | .246 | .474** | .426** | .460** | .435** | 1 |  |
| MPFI_CF | .562** | .697** | .623** | .472** | .572** | .638** | .471** | .423** | .473** | .716** | 1 |
| **Cognitive inflexibility (MPFI_CINF)** | | | | | | | | | | | |
|  | MPFI11 | MPFI12 | MPFI13 | MPFI14 | MPFI15 | MPFI16 | MPFI17 | MPFI18 | MPFI19 | MPFI20 | MPFI_CINF |
| MPFI11 | 1 |  |  |  |  |  |  |  |  |  |  |
| MPFI12 | .764** | 1 |  |  |  |  |  |  |  |  |  |
| MPFI13 | .656** | .749** | 1 |  |  |  |  |  |  |  |  |
| MPFI14 | .564** | .468** | .619** | 1 |  |  |  |  |  |  |  |
| MPFI15 | .590** | .674** | .623** | .659** | 1 |  |  |  |  |  |  |
| MPFI16 | .495** | .490** | .330* | .357* | .528** | 1 |  |  |  |  |  |
| MPFI17 | .477** | .491** | .368* | .459** | .572** | .851** | 1 |  |  |  |  |
| MPFI18 | .404** | .499** | .348* | .382** | .655** | .730** | .696** | 1 |  |  |  |
| MPFI19 | .345* | .369* | .299* | .334* | .437** | .722** | .661** | .572** | 1 |  |  |
| MPFI20 | .306* | .358* | .333* | .487** | .421** | .715** | .785** | .614** | .625** | 1 |  |
| MPFI_CINF | .740** | .774** | .699** | .702** | .809** | .818** | .837** | .776** | .701** | .745** | 1 |

2. **ORGANIZATIONAL FACTORS**

- **Social Support**

| **Social Support - Friends (SS)** | | | |  |  |  |
| --- | --- | --- | --- | --- | --- | --- |
|  | SS1 | SS2 | SS_Friends |  |  |  |
| SS1 | 1 |  |  |  |  |  |
| SS2 | .650** | 1 |  |  |  |  |
| SS_Friends | .897** | .919** | 1 |  |  |  |
| **Social Support - Family (SS)** | | | |  |  |  |
|  | SS3 | SS4 | SS_Family |  |  |  |
| SS3 | 1 |  |  |  |  |  |
| SS4 | .702** | 1 |  |  |  |  |
| SS_Family | .918** | .926** | 1 |  |  |  |
| **Social Support - Supervisor/Manager (SS)** | | | | |  |  |
|  | SS5 | SS6 | SS7 | SS_S/M |  |  |
| SS5 | 1 |  |  |  |  |  |
| SS6 | .753** | 1 |  |  |  |  |
| SS7 | .691** | .775** | 1 |  |  |  |
| SS_S/M | .906** | .929** | .891** | 1 |  |  |
| **Social Support - Colleagues (SS)** | | | | |  |  |
|  | SS8 | SS9 | SS10 | SS_Coll |  |  |
| SS8 | 1 |  |  |  |  |  |
| SS9 | .709** | 1 |  |  |  |  |
| SS10 | .621** | .656** | 1 |  |  |  |
| SS_Coll | .874** | .892** | .874** | 1 |  |  |
| **Social Support - Organization (SS)** | | | | |  |  |
|  | SS11 | SS12 | SS13 | SS_ORG |  |  |
| SS11 | 1 |  |  |  |  |  |
| SS12 | .838** | 1 |  |  |  |  |
| SS13 | .845** | .809** | 1 |  |  |  |
| SS_ORG | .954** | .931** | .940** | 1 |  |  |

- **Organizational resilience scale (SEOR)**

| **Skill-based resilience (SEOR_SR)** | | | | | | |  |
| --- | --- | --- | --- | --- | --- | --- | --- |
|  | SEOR1 | SEOR2 | SEOR3 | SEOR4 | SEOR5 | SEOR6 | SEOR_SR |
| SEOR1 | 1 |  |  |  |  |  |  |
| SEOR2 | .817** | 1 |  |  |  |  |  |
| SEOR3 | .779** | .729** | 1 |  |  |  |  |
| SEOR4 | .703** | .696** | .713** | 1 |  |  |  |
| SEOR5 | .594** | .651** | .756** | .669** | 1 |  |  |
| SEOR6 | .674** | .725** | .806** | .696** | .871** | 1 |  |
| SEOR_SR | .873** | .876** | .913** | .843** | .858** | .904** | 1 |
| **Individual-based resilience (SEOR_IR)** | | | | | | |  |
|  | SEOR7 | SEOR8 | SEOR9 | SEOR_IR |  |  |  |
| SEOR7 | 1 |  |  |  |  |  |  |
| SEOR8 | .609** | 1 |  |  |  |  |  |
| SEOR9 | .523** | .530** | 1 |  |  |  |  |
| SEOR_IR | .840** | .854** | .820** | 1 |  |  |  |
